# On the optimal design of metabolic RNA labeling experiments

**DOI:** 10.1371/journal.pcbi.1007252

**Published:** 2019-08-07

**Authors:** Alexey Uvarovskii, Isabel S. Naarmann-de Vries, Christoph Dieterich

**Affiliations:** 1 Klaus Tschira Institute for Integrative Computational Cardiology and Department of Internal Medicine III, University Hospital Heidelberg, Heidelberg, Germany; 2 German Center for Cardiovascular Research (DZHK), Partner site Heidelberg-Mannheim, Heidelberg, Germany; 3 Department of Intensive Care Medicine, University Hospital Aachen, RWTH Aachen University, Aachen, Germany; Julius-Maximilians-Universitat Wurzburg, GERMANY

## Abstract

Massively parallel RNA sequencing (RNA-seq) in combination with metabolic labeling has become the *de facto* standard approach to study alterations in RNA transcription, processing or decay. Regardless of advances in the experimental protocols and techniques, every experimentalist needs to specify the key aspects of experimental design: For example, which protocol should be used (biochemical separation vs. nucleotide conversion) and what is the optimal labeling time? In this work, we provide approximate answers to these questions using the asymptotic theory of optimal design. Specifically, we investigate, how the variance of degradation rate estimates depends on the time and derive the optimal time for any given degradation rate. Subsequently, we show that an increase in sample numbers should be preferred over an increase in sequencing depth. Lastly, we provide some guidance on use cases when laborious biochemical separation outcompetes recent nucleotide conversion based methods (such as SLAMseq) and show, how inefficient conversion influences the precision of estimates. Code and documentation can be found at https://github.com/dieterich-lab/DesignMetabolicRNAlabeling.

## Author summary

Massively parallel RNA sequencing (RNA-seq) in combination with metabolic labeling has become the *de facto* standard approach to study alterations in RNA transcription, processing or decay. In our manuscript, we address several key aspects of experimental design: 1) The optimal labeling time, 2) the number of replicate samples over sequencing depth and 3) the choice of experimental protocol. We provide approximate answers to these questions using asymptotic theory of optimal design.

## Introduction

Changes in gene expression are frequently observed in pathological conditions. In the simplest model [1], steady state RNA levels are governed by synthesis (transcription) and degradation rates (RNA stability). A paradigm is the generation of the hypoxic response in pathological conditions such as heart insufficiency [2] and fast growing tumors [3]. Hypoxia (<2% O_2_) results in a global decrease of total transcription [4]. However, the transcription of specific target genes is induced under hypoxic conditions by hypoxia inducible factor 1 (HIF1) [5], which is composed of a stable *β*-subunit and an oxygen labile *α*-subunit [6]. Furthermore, different RNA binding proteins such as HuR and TTP as well as miRNAs regulate the stability of their cognate target mRNAs dependent on oxygen availability [7] and contribute to changes in gene expression profiles.

Metabolic labeling experiments are a versatile tool to discern dynamic aspects in physiological and pathological processes. These experiments drive our understanding of key processes in molecular systems, such as synthesis and decay of metabolites, DNA, RNA and proteins. Pulse-chase experiments help to determine the kinetic parameters of synthesis and decay in various contexts. In the pulse phase of an experiment, the label is introduced to newly synthesized compounds and unlabeled or pre-existing molecules are only subjected to degradation or some other form of processing. In contrast, during the chase phase, the label in the system is gradually replaced by unlabeled compounds. A typical metabolic labeling experiment may include a pulse, a chase or both phases.

The first transcriptome-wide studies by [8] and [9] used 4-thiouridine (4sU) labeling in cell culture experiments to infer kinetic parameters. This approach has become quite popular in RNA biology, which is shown by a vastly increasing number of studies (see [10] for review).

Massively parallel RNA sequencing (RNA-seq) in combination with metabolic labeling has become the *de facto* standard approach to study alterations in RNA transcription, processing or decay at the transcriptome-wide level. At the time of writing, the most widely used approach involves metabolic labeling with thiol-labeled nucleoside analogs such as 4sU (4sU-tagging) [11]. Briefly, total cellular RNA is isolated and thiol groups are biotinylated. Subsequently, total cellular RNA can be efficiently separated into newly transcribed (labeled) and pre-existing (unlabeled) RNA.

Very recent innovations are new methods involving the chemical conversion of 4sU residues into cytosine analogs, which is observed as point mutations in RNA-seq data (T-to-C transitions), (see [12], [13] and [14]). The absence of any biochemical separation method makes metabolic labeling more accessible due to lower input amounts and less laborious protocols.

Regardless of all advances in the experimental protocols and techniques, a few important questions remain to be answered by any experimentalist, namely the specific characteristics of experimental design: what should be measured (i.e. sequenced) and when? For example, which approach should I take (e.g. biochemical separation vs. nucleotide conversion), when should I collect my samples (e.g. time points in a pulse experiment) and how could this affect my estimates on kinetic parameters. In [15], the authors proposed guidelines for the design of metabolic labeling experiments, however they provide no kinetic or statistical models for the optimization of such experiments.

**Fig 1:**
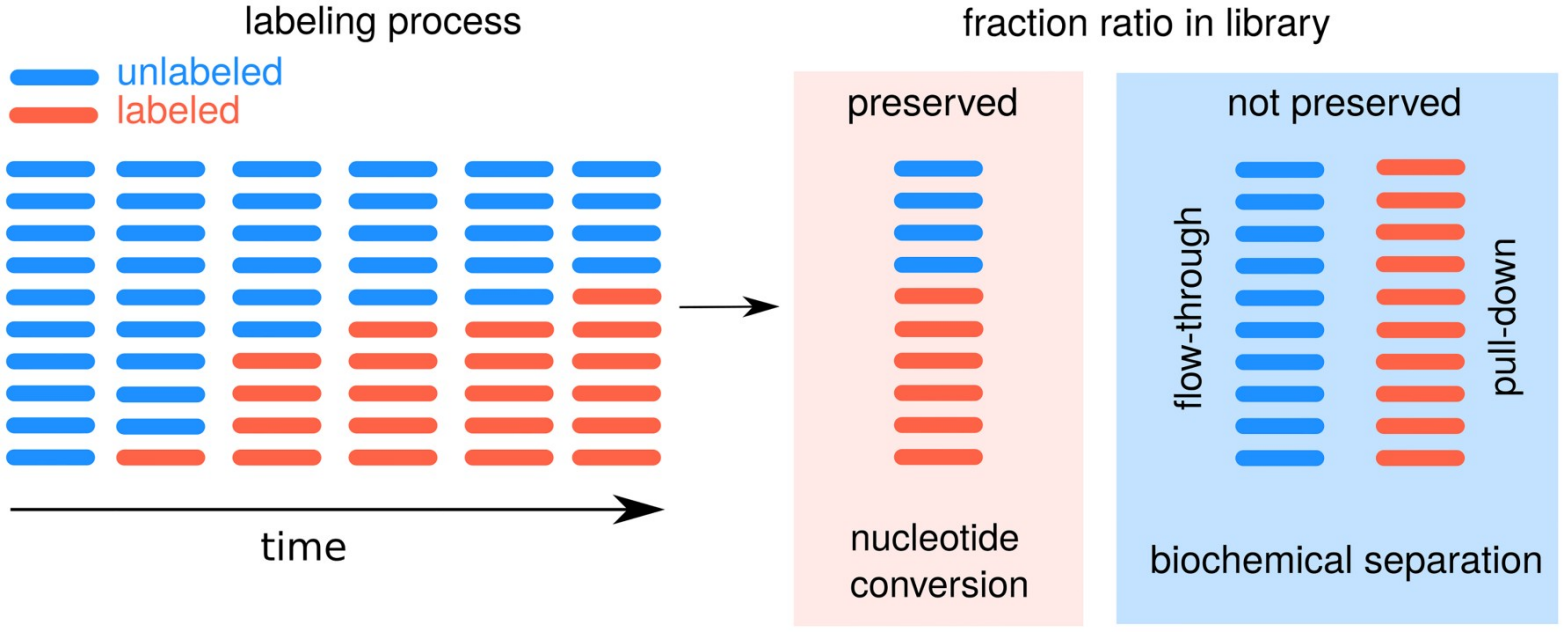
Pulse labeling experiment types to measure degradation rates. The conventional approach as in [18] utilizes biochemical separation, which does not preserve the fraction ratio (labeled vs. unlabeled) in the read counts. Alternative novel approaches (e.g. [12]) induce reverse transcription signature events (nucleotide conversions, typically T-to-C). Individual reads can be classified by the presence or absence of this characteristic nucleotide conversions. In an ideal case, the fraction ratio is well reflected by the read counts, however in practice a relatively low 4sU incorporation rate of 1:40 has to be taken into account ([12], [9]).

Within this manuscript, we use kinetic and statistical models to infer the degradation rates from a pulse experiment (see Fig 1 and Eqs 1 and 2), and derive several aspects on the optimal design of metabolic RNA labeling experiments. We illustrate these implications on a pulse-chase SLAMseq data set [12] and an example for a pulse labeling experiment with biochemical separation.

## Materials and methods

### Tissue culture cell line

MCF-7 cells (ACC-115) were obtained from the Leibniz Institute DSMZ German Collection of Microorganisms and Cell Cultures. Cells were routinely tested for mycoplasma contamination with Venor GeM Classic (Minerva Biolabs). MCF-7 cells were cultured at 37°C and 5% CO_2_ and maintained in DMEM (Thermo Fisher Scientific) supplemented with 10% fetal calf serum (Merck), 1xMEM non-essential amino acids (Thermo Fisher Scientific) and 1xPenicillin/Streptomycin (Thermo Fisher Scientific).

### Tissue culture

MCF-7 cells were seeded 48 hrs prior to the experiment at a cell density of 0.3 × 10^5^cells/cm^2^. Cells were labeled with 4-thiouridine (4sU) (Sigma-Aldrich) at a final concentration of 200 *μ*M for 2, 4 or 8 hrs. Cells were scraped in DPBS and the pellet resuspended in Trizol (Thermo Fisher Scientific).

### Isolation of total RNA

Total RNA was isolated using the Trizol method. Briefly, the cell pellet was resuspended in 750 *μ*l Trizol, and incubated 5 min at room temperature before addition of 200 *μ*l chloroform. Samples were centrifuged (20 min, 10.000g, room temperature) and the aqueous phase re-extracted with one volume chloroform: isoamylalkohol (24:1) (5 min, 10.000g, room temperature). The RNA in the aqueous phase was precipitated with one volume isopropanol (30 min, 20.8000g, 4°C), washed twice with 1 ml 80% ethanol in DEPC-H_2_O and dissolved in 25 *μ*l DEPC-H_2_O (10 min, 55°C, shaking).

### *In vitro* transcription of spike ins

For *in vitro* transcription of linearized plasmids (pBSIIKS-Luc-pA-NB [16] and pBSIIKS-Renilla-pA [17]), the MEGAscript T7 Transcription Kit (Thermo Fisher Scientific) was used according to the manufacturers instructions. Briefly, the reaction was set up in a total volume of 20 *μ*l containing 1 *μ*g linearized plasmid and 2 *μ*l 10x reaction buffer, 3 *μ*l 40 mM m^7^GppG-cap analogon (KEDAR), 2 *μ*l 15 mM GTP, 2 *μ*l 75 mM CTP, 2 *μ*l 75 mM ATP, 2 *μ*l enzyme mix and 2 *μ*l 75 mM UTP (for RLuc) or 2 *μ*l 75 mM 4-S-UTP:UTP in a 1:10 ratio (for FLuc). Reactions were incubated 3 hrs at 37°C. Plasmid-DNA was removed by addition of 1 *μ*l Turbo-DNase (15 min, 37°C). *In vitro* transcribed RNA was purified by phenol extraction and Chromaspin-100 (Clontech) purification. RNA was precipitated over night after addition of sodium acetate to a final concentration of 0.3 M and 2.5 volumes 100% ethanol. After centrifugation (30 min, 20.800g, 4°C) the pellet was washed with 1 ml 80% ethanol and dissolved in 40 *μ*l DEPC-H_2_O. Concentration was determined by Nanodrop (Thermo Fisher Scientific) measurement and integrity checked by agarose gel electrophoresis.

### Biotinylation of RNA

Total RNA was spiked with *in vitro* transcribed 4sU-labeled FLuc and non-labeled RLuc RNAs and biotinylated using MTSEA biotin-XX (Biotium) as described by [18]. Briefly 80 *μ*g total RNA was incubated with 8 ng FLuc and 4.8 ng RLuc (equimolar amounts, 130 amol), 10 mM HEPES pH 7.5, 1 mM EDTA and 5 *μ*g MTSEA biotin-XX (freshly dissolved in DMF) in a total volume of 250 *μ*l. Reactions were incubated 30 min in the dark at room temperature. Biotinylated RNA was recovered by extraction with one volume phenol: chloroform: isoamylalkohol (24:24:1) and separated using Phase-Lock-tubes (5Prime) by centrifugation (5 min, 20.800g, room temperature). RNA was precipitated by addition of 350 *μ*l isopropanol, 25 *μ*l 5 M sodium chloride and 1 *μ*l glycogen (Roche Diagnostics, 20 *μ*g/*μ*l) to assist precipitation (30 min, 20.800g, 4°C). RNA was washed twice with 500 *μ*l 80% ethanol in DEPC-H_2_O and dissolved in 25 *μ*l DEPC-H_2_O (10 min, 55°C, shaking).

### Streptavidin purification

For purification of biotinylated RNAs the method described by [1] was adapted. 25 *μ*g biotinylated total RNA was adjusted to 100 *μ*l with DEPC-H_2_O and filled up with Streptavidin binding buffer (Strep-BB) (20 mM Tris, pH 7.4, 0.5 M sodium chloride, 1 mM EDTA) to 200 *μ*l. RNA was denatured 10 min at 65°C and subsequently placed on ice. 100 *μ*l magnetic streptavidin beads (New England Biolabs) were washed once with 200 *μ*l Strep-BB and resuspended in 100 *μ*l Strep-BB. RNA and beads were incubated 15 min at room temperature on a rotating wheel. Beads were washed three times with 500 *μ*l Strep washing buffer (100 mM Tris pH 7.4, 1 M sodium chloride, 10 mM EDTA, 0.1% Tween 20) prewarmed to 55°C. RNA was eluted three times by de-biotinylation with 100 *μ*l freshly prepared 100 mM DTT and elution fractions pooled for further analysis. RNA was recovered from total RNA, flow through and eluate by phenol: chloroform: isoamylalkohol (24:24:1) extraction using Phase-Lock-tubes and isopropanol precipitation as described above. The amount of recovered RNA was determined by Nanodrop measurement.

### Dot blot-based detection of biotinylation

1 *μ*g biotinylated RNA was applied to nylon membrane (Hybond-N, GE Healthcare) using a dot blot device (Carl Roth). RNA was crosslinked twice at 254 nm using the “Optimal Crosslink” mode of the Spectroline Select XLE-1000 crosslinker. The membrane was blocked 20 min with PBS + 10% SDS and incubated 2 hrs with Streptavidin-HRP (Thermo Fisher Scientific, 1:5000 in PBS + 10% SDS). Prior to detection with SuperSignal West Pico (Thermo Fisher Scientific) the membrane was washed each three times 10 min with PBS + 10% SDS, PBS + 1% SDS and PBS + 0.1% SDS. Images were acquired with the LAS4000 system (GE Healthcare).

### Reverse transcription

1 *μ*l RNA from streptavidin purification was reverse transcribed using the Maxima H Minus First Strand cDNA Synthesis Kit (Thermo Fisher Scientific) with Random Primers according to the manufacturers protocol. For absolute quantification reverse transcription reactions were set up with different amounts of spike in RNAs, ranging from 1600% to 1.56% for FLuc and 400 to 3.12% for RLuc in 1:2 dilutions. Briefly, RNA was mixed in a total volume of 15 *μ*l with 1 *μ*l Random Primer and 1 *μ*l dNTP solution and denatured (5 min, 65°C). Reaction was completed by addition of 4 *μ*l 5xRT buffer and 1 *μ*l Maxima enzyme and incubated 10 min at room temperature followed by 30 min, 50°C and denaturation (5 min, 85°C).

### qPCR analysis

Reverse transcription reactions were diluted 1:10 and used for qPCR analysis on a StepOnePlus instrument (ThermoFisherScientific) with Power SYBR Green PCR Master Mix (Thermo Fisher Scientific) and primers directed against FLuc (forward: CCTTCCGCATAGAACTGCCT, reverse: GGTTGGTACTAGCAACGCAC [19]) and RLuc (forward: GTTGTGCCACATATTGAGCC, reverse: CCAAACAAGCACCCCAATCATG [20]).

### Sequencing

Total and enriched samples were depleted for ribosomal RNA (rRNA) contamination using RiboZeroGold, which is based on the removal of rRNA with biotinylated oligos using streptavidin beads. Thus, also the biotinylated 4sU-labeled molecules were removed from the total samples by the RiboZeroGold procedure and were treated as flow through. Libraries of 2 biological replicate 4sU pulse experiment were sequenced 1x 50bp on an Illumina HiSeq4000. All relevant details on sequencing depth and mapping rates are listed in S1 Table.

### Read processing and counting

Sequencing adapters and low-quality reads were removed from the raw sequencing data with flexbar v3.0.3 [21] using standard filtering parameters. We excluded all reads with more than 1 uncalled base from the output. All remaining reads (>18bp) were then aligned to a custom sequence index including rRNA, tRNA and snoRNA gene loci using bowtie2 with the –very-fast option [22]. Only reads that did not align to any of the contaminant sequences were considered for further analysis.

Reads were then aligned to the human genome (EnsEMBL 85) and splice sites from the reference annotation with a splice-aware aligner (STAR, v2.5.3a; [23]). The BAM files were analyzed with StringTie 1.3.3b [24] and the final read count matrix was prepared with the supplemented python script prepDE.py.

## Results

### Model of the experiment

We describe RNA-seq read counts with the negative binomial distribution, which is widely used in this setting and accounts for overdispersion [25]. For a given gene, the read count follows *X* ∼ *NB*(*m*(*μ*, *δ*, *t*), *k*), where *m* is the mean read count, which depends on the time of labeling *t*, the degradation rate *δ* and the expression level in the steady-state *μ*, and *k* is the overdispersion parameter of the negative binomial distribution *NB*. In this case, the variance is var(*X*) = *m*(*m* + *k*)/*k*, where low *k* values correspond to high overdispersion in the data.

We describe the RNA amount *m* in metabolic labeling experiments using simple first order kinetics:

$$\begin{array}{c} \frac{\text{d}m}{\text{d}t}=s-\delta m, \end{array}$$ (1)

where *s* is the synthesis rate and *δ* is the degradation rate. In a steady-state, the expression level of a gene is *μ* = *s*/*δ*. The expression level *μ* can be derived from the total fraction, which ensures identifiability of at least this parameter. For that reason, we use *μ* and *δ* to parametrize the model. In this section, we only discuss the case of pulse labeling experiments throughout. However, our considerations extend to chase labeling experiments, where the equations are the same, except that the labeled fraction behaves as the unlabeled one in the pulse experiment and *vice versa*. For simplicity, we assume that fraction cross-contamination is negligible, in which case, RNA amounts for a given gene are proportional to the means *m*_*L*_, *m*_*U*_ and *m*_*T*_ derived from the kinetics for labeled, unlabeled and total fractions scaled by sample-specific factors *x*_i_ (see Eq 4 in section 2 of Extended Methods):

$$\begin{array}{c} \begin{array}{cc} m_{\text{T}}\left(t\right) & =1\cdot \mu \\ m_{\text{L}}\left(t\right) & =x_{\text{L}}\mu \left(1-e^{-\delta t}\right)\\ m_{\text{U}}\left(t\right) & =x_{\text{U}}\mu e^{-\delta t} \end{array} \end{array}$$ (2)

Here we treat the mean read count in the total sample as a reference (coefficient is 1), to make the system identifiable. In the case of labeled and unlabeled fractions, expected read numbers must be scaled by additional coefficients, *x*_U_ and *x*_L_, because the RNA material can be normalized by different degrees during library preparation from chemically separated fractions.

A preservation of the ratio of labeled to unlabeled fractions (see Fig 1) yields *x*_U_ = *x*_L_. If the sequencing depth is approximately the same for all samples, we may assume for simplicity *x*_U_ = *x*_L_ = 1, and in this case, *m*_T_(*t*) = *m*_L_(*t*) + *m*_U_(*t*) = *μ*.

In the conventional approach, where labeled and unlabeled molecules are separated, *x*_U_ ≠ *x*_L_, the fraction ratio must be inferred from the data itself or by using an external normalization by spiking in labeled and unlabeled known molecules [26]. In the presence of cross-contamination, the estimations for the rates are biased depending on the relation of the labeling time and the degradation rate: if *δt* ≪ 1 (slow rate), the bias is towards faster rate values, and, if *δt* ≫ 1 (fast rate), it is towards slower rate values, for more details see Eqs 13 and 14, section 2.1 in Extended methods. Efficiency of separation procedure may vary between species due to different uridine content, which can be another source of bias, see section 2.2 in Extended methods. This phenomenon can be modeled by introducing an additional coefficient to the model, see, for example, [27] and [28]. Although both sources of a bias may potentially affect estimates of certain RNA species, they are beyond the scope of our current work. Here, we concentrate on theoretical results, which are derived from statistical properties of our outlined model.

### The best time to measure

In the following, we discuss pulse labeling experiments with different labeling times *t*. On the one hand, subtle changes in the RNA level are masked by the measurement noise for short labeling times. On the other hand, estimations at long labeling times are also less informative, because the difference between the steady state level and the RNA levels at time *t* is negligible and will be masked by the noise as well.

To estimate the degradation rate *δ* from the RNAseq read counts, we use the method of maximum likelihood estimation (MLE). This estimator $\hat{\delta }$ varies from experiment to experiment, and one is interested to minimize its variance, as a large variance results in large confidence intervals and, hence, poor estimates of the true *δ*. In this paper, we use the asymptotic properties of the MLE, when the number of experiment repetitions *n* → ∞, in which case the system can be treated analytically [29, 30].

Under regularity conditions, the MLE $\hat{\theta }$ is asymptotically normally distributed:

$$\begin{array}{c} \sqrt{n}\left(\hat{\theta }-\theta \right)∼N(0,I_{1}^{-1}\left(\theta \right)), \end{array}$$ (3)

where $I_{1}\left(\theta \right)$ is the Fisher information matrix (FIM) for a single experiment repetition [29, 30].

The FIM characterizes the curvature of the log-likelihood function L(θ,X) near the true parameter values ***θ*** and is defined as

$$\begin{array}{c} I_{\text{ij}}\left(\theta \right)=-E\frac{\partial^{2}\log L\left(\theta ,X\right)}{\partial \theta_{i}\partial \theta_{j}}. \end{array}$$ (4)

We assume that the overdispersion parameter *k* is shared between all genes and neglect the uncertainty in *δ* propagating from *k*, i.e. only two parameters, *δ* and *μ*, are used to construct the FIM:

$$\begin{array}{c} I\left(\theta \right)=(\begin{array}{cc} I_{\delta \delta }\left(\theta \right) & I_{\delta \mu }\left(\theta \right)\\ I_{\delta \mu }\left(\theta \right) & I_{\mu \mu }\left(\theta \right). \end{array}) \end{array}$$ (5)

The FIM is additive, i.e. if $I_{\text{U}}\left(\theta \right)$ and $I_{\text{L}}\left(\theta \right)$ correspond to the labeled and unlabeled fractions, the total FIM for the experiment is $I\left(\theta \right)=I_{\text{U}}\left(\theta \right)+I_{\text{L}}\left(\theta \right)$, and for *n* such repetitions, $I\left(\theta \right)=n(I_{\text{U}}\left(\theta \right)+I_{\text{L}}\left(\theta \right))$.

The diagonal terms of the inverse FIM estimate the variance of $\hat{\theta_{i}}$

$$\begin{array}{c} \text{var}\left(\hat{\theta_{i}}\right)={\left(I^{-1}\left(\theta \right)\right)}_{\text{ii}}. \end{array}$$ (6)

In some cases we use $1/I_{\text{ii}}\left(\theta \right)$ as a lower bound for ${\left(I^{-1}\left(\theta \right)\right)}_{\text{ii}}$. Since

$$\begin{array}{c} {\left(I^{-1}\left(\theta \right)\right)}_{\delta \delta }={\left(I_{\delta \delta }\left(\theta \right)-I_{\delta \mu }\left(\theta \right)I_{\mu \delta }\left(\theta \right)/I_{\mu \mu }\left(\theta \right)\right)}^{-1}, \end{array}$$ (7)

and using the fact that $I_{\delta \mu }\left(\theta \right)=I_{\mu \delta }\left(\theta \right)$ and $I_{\mu \mu }\left(\theta \right)>0$, the diagonal term of the inverse matrix is bounded as

$$\begin{array}{c} {\left(I^{-1}\left(\theta \right)\right)}_{\delta \delta }⩾1/I_{\delta \delta }\left(\theta \right). \end{array}$$ (8)

${\left(I^{-1}\left(\theta \right)\right)}_{\delta \delta }=1/I_{\delta \delta }\left(\theta \right)$ if there is no uncertainty, propagating from other parameters, i.e. $I_{\delta \mu }\left(\theta \right)=0$.

Since the FIM I(θ) depends on the experiment parameters, such as the labeling time *t* and the sequencing depth, it is our main interest to reduce the variance of the MLE by selecting the optimal conditions accordingly. Due to additive property of the FIM, it suffices to optimize the FIM of a single experiment repetition.

In the case of multiple parameters, it may be not possible to achieve the minimal variance for all parameters at the same time. Different criteria can be constructed as a combination of the elements of the inverse FIM [29, 31]. We are interested to optimize the estimation of *δ* only and do not consider variance of the expression level estimator $\hat{\mu }$ in the design criteria.

Let us consider first a simpler experimental setup, which preserves the fraction ratio (e.g. SLAMseq). Here we first discuss the case of the Poisson model, which corresponds to the case of no overdispersion (*k* → ∞). The derivations for the Poissonian and for more general cases are left to section 3 of the Extended Methods, see Eqs 25 and 26. Let *X*_L_ and *X*_U_ be the read counts corresponding to the labeled and unlabeled molecules for a given gene in a SLAMseq sample, and let *t* be the time of labeling. In this case, the inverse FIM is diagonal:

$$\begin{array}{c} I_{\text{slam}}^{-1}\left(\theta \right)={\left(I_{\text{L}}\left(\theta \right)+I_{\text{U}}\left(\theta \right)\right)}^{-1}=(\begin{array}{cc} \frac{e^{\delta t}-1}{\mu t^{2}} & 0\\ 0 & \mu \end{array}) \end{array}$$ (9)

The parameters *δ* and *μ* are information orthogonal, because $I_{\delta \mu }\left(\theta \right)=0$ and inference about *δ* can be done as *μ* were known exactly.

Indeed, for *X*_L_ ∼ Pois(*m*_L_(*t*)), *X*_U_ ∼ Pois(*m*_U_(*t*)), the conditional distributions *P*(*X*_L_|*X*_U_ + *X*_L_) and *P*(*X*_U_|*X*_U_ + *X*_L_) are binomial with the rates *m*_U_(*t*)/(*m*_U_(*t*) + *m*_L_(*t*)) = *e*^−*δt*^ and *m*_L_(*t*)/(*m*_U_(*t*) + *m*_L_(*t*)) = 1 − *e*^−*δt*^ and do not depend on *μ*. This model was recently discussed in a Bayesian framework for SLAMseq experiments by [32].

For a diagonal I(θ), the inverse term ${\left(I_{\text{slam}}^{-1}(\theta )\right)}_{\delta \delta }={\left(\left(I_{\text{slam}}(\theta \right)\right)}_{\delta \delta }{)}^{-1}={\left(\left(I_{\text{U}}(\theta \right)\right)}_{\delta \delta }+{\left(I_{\text{L}}(\theta )\right)}_{\delta \delta }{)}^{-1}.$ The maximum of the term ${\left(I_{\text{slam}}\left(\theta \right)\right)}_{\delta \delta }$ corresponds to the minimal asymptotic variance of $\hat{\delta }$ due to Eq 3. By optimizing ${\left(I_{\text{slam}}\left(\theta \right)\right)}_{\delta \delta }$ with respect to *t*, we get

$$\begin{array}{c} t_{\text{slam}}=1.59\tau ,\; \end{array}$$ (10)

where *τ* = 1/*δ* is the characteristic time of degradation. That means, if one optimizes the SLAMseq experiment and targets the gene with the characteristic time of degradation *τ*, the measurement at time point 1.59*τ* corresponds to the asymptotically optimal design. For example, if one is interested in an RNA species with half-life time of λ = 1 hr (i.e. the characteristic time *τ* = λ/log(2) ≈ 1.44 hr), a pulse phase of 1.59 × 1.44 ≈ 2.3 hr corresponds to the asymptotically optimal design.

**Fig 2:**
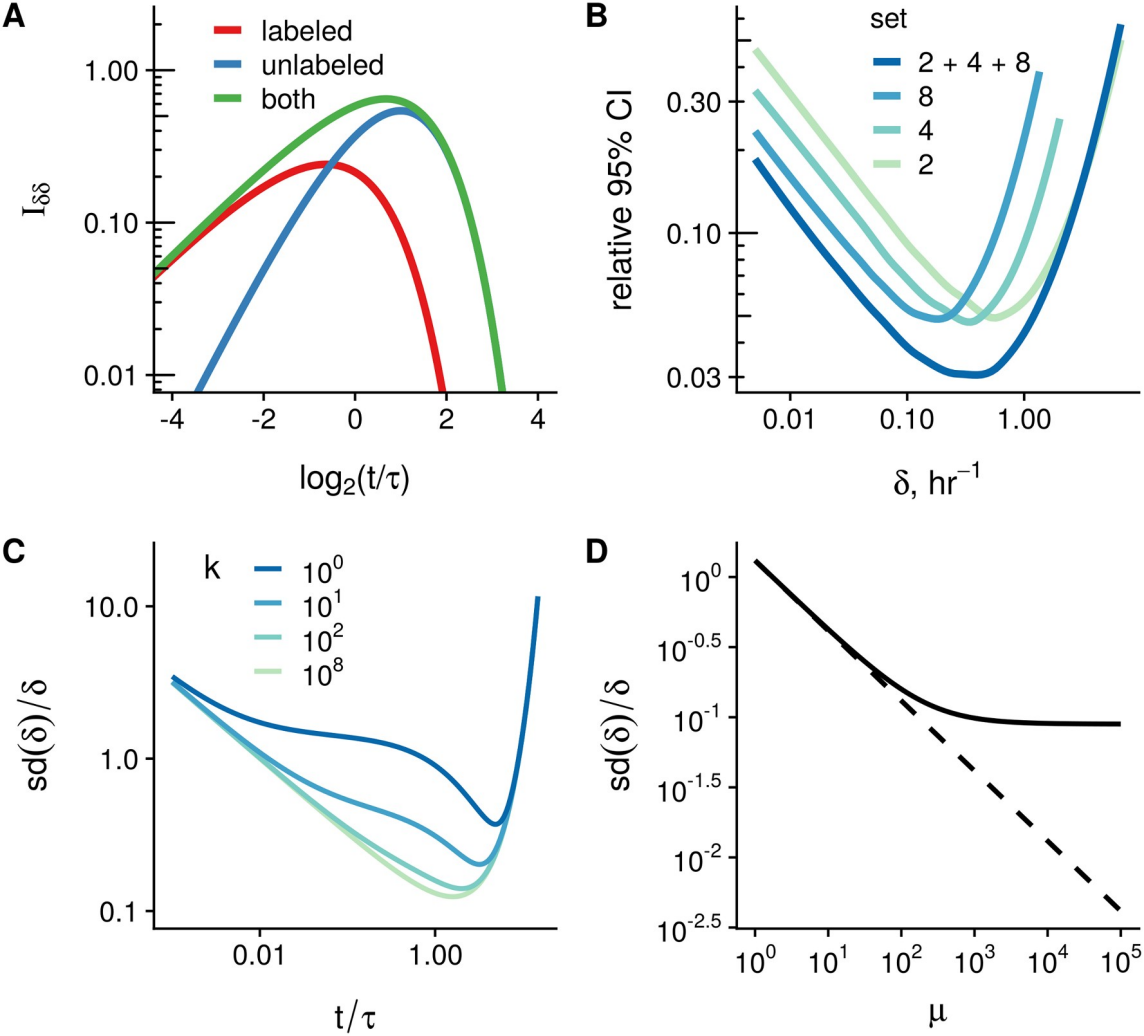
The key characteristics of metabolic RNA labeling experiments. **A**: The diagonal term of the Fisher information matrix (FIM) $I_{\delta \delta }\left(\theta \right)$, as a function of the ratio of labeling time *t* to the characteristic time of degradation *τ* = 1/*δ* for the case of SLAMseq experiment. Read counts follow the Poisson distribution, the expression level is *μ* = 1 and the degradation rate is *δ* = 1. **B**: 95% confidence interval (CI) relative width of the degradation rates for different sets of time points included in the simulation of the SLAMseq experiment. We simulated counts for a range of rates *δ* and assumed for simplicity that normalization factors are perfectly known but not the rates and expression levels. Smoothed data from 10 simulation runs is shown. **C**: Relative standard deviation ($sd(\hat{\delta })/\delta$) of the MLE for *δ* as a function of measurement time at different values of the overdispersion parameter *k*. With increasing overdispersion, the profile of the dependency flattens. However, near the optimal time point, variance of the estimation is more sensitive to time of labeling, which complicates the optimal design choice for different *δ* ranges. Expression level is fixed to *μ* = 100 reads in this example, the degradation rate is assumed to be *δ* = 1. The FIM $I\left(\theta \right)=nI_{1}\left(\theta \right)$ is calculated for *n* = 1. **D**: Relative standard deviation ($sd(\hat{\delta })/\delta$) for a model with overdispersion (*k* = 100, solid line) or with no overdispersion (*k* → ∞, dashed line). The degradation rate is *δ* = 1, the labeling time is *t* = 1. The FIM $I\left(\theta \right)=nI_{1}\left(\theta \right)$ is calculated for *n* = 1.

In Fig 2A, we depicted the dependency of ${\left(I_{\text{slam}}\left(\theta \right)\right)}_{\delta \delta }$ and corresponding values of ${\left(I_{\text{U}}\left(\theta \right)\right)}_{\delta \delta }$ and ${\left(I_{\text{L}}\left(\theta \right)\right)}_{\delta \delta }$ as functions of normalized time *t*/*τ* for the degradation rate *δ* = 1. Interestingly, ${\left(I_{\text{U}}\left(\theta \right)\right)}_{\delta \delta }$ and ${\left(I_{\text{L}}\left(\theta \right)\right)}_{\delta \delta }$ achieve maximum at *t*_*U*_ = 2*τ* and *t*_*L*_ ≈ 0.64*τ*, and the main contribution to the sum ${\left(I_{\text{slam}}(\theta )\right)}_{\delta \delta }={\left(I_{\text{U}}(\theta )\right)}_{\delta \delta }+{\left(I_{\text{L}}(\theta )\right)}_{\delta \delta }$ comes from the term corresponding to labeled counts at shorter labeling times, and from the term for unlabeled counts at times longer than *τ*, see Fig 2A.

### Cost of suboptimal timing

Usually one is interested to measure a rate with a certain relative precision. To reflect this, we normalize the variance of the degradation rate estimator by *δ*^2^:

$$\begin{array}{c} \frac{\text{var}(\hat{\delta })}{\delta^{2}}\approx \frac{1}{I_{\delta \delta }\left(\theta \right)\delta^{2}}. \end{array}$$ (11)

Using a non-dimensional substitute *α* = *t*/*τ*, the corresponding denominator terms are

$$\begin{array}{c} \begin{array}{cc} {\left(I_{\text{L}}\left(\theta \right)\right)}_{\delta \delta }\delta^{2} & =\frac{\alpha^{2}\mu }{e^{2\alpha }-e^{\alpha }}\\ {\left(I_{\text{U}}\left(\theta \right)\right)}_{\delta \delta }\delta^{2} & =\alpha^{2}e^{-\alpha }\mu \\ {\left(I_{\text{slam}}\left(\theta \right)\right)}_{\delta \delta }\delta^{2} & =\frac{\alpha^{2}\mu }{e^{\alpha }-1}, \end{array} \end{array}$$ (12)

see Eqs 50, section 3.5 in Extended Methods.

For labeling times much shorter than the characteristic degradation time of a given gene, *α* ≪ 1, the normalized FIM terms behave as a power function:

$$\begin{array}{c} {\left(I_{\text{slam}}\left(\theta \right)\right)}_{\delta \delta }\delta^{2},{\left(I_{\text{L}}\left(\theta \right)\right)}_{\delta \delta }\delta^{2}∼\alpha ,\;{\left(I_{\text{U}}\left(\theta \right)\right)}_{\delta \delta }\delta^{2}∼\alpha^{2}. \end{array}$$ (13)

However, for labeling times much longer than the characteristic time of degradation *τ*, *α* ≫ 1, the normalized FIM terms vanish exponentially:

$$\begin{array}{c} {\left(I_{\text{L}}\left(\theta \right)\right)}_{\delta \delta }\delta^{2}∼e^{-2\alpha },\;{\left(I_{\text{slam}}\left(\theta \right)\right)}_{\delta \delta }\delta^{2},{\left(I_{\text{U}}\left(\theta \right)\right)}_{\delta \delta }\delta^{2}∼e^{-\alpha }, \end{array}$$ (14)

see derivations in Extended Methods, section 3.5, Eqs 51 and 52.

In a typical high-throughput experiment, the kinetic parameters are monitored for a large set of genes (in the order of thousands), which may have different degradation rates. In this case, every time point in the experiment will be only optimal for a subset of these genes. To illustrate this effect, we simulated read counts for an ideal SLAMseq experiment (with no overdispersion) and fitted the model using various sets of samples. In our *in silico* experiment, we always included the total fraction (*t* = 0 hr), and either one additional time point (labeled and unlabeled fractions) or all time points (2, 4, and 8 hr). The normalization coefficients were set to 1 to mimic an ideal SLAMseq scheme, as discussed earlier, Eq 2.

We fitted the model using the pulseR package and computed the 95% confidence intervals (CI) for *δ* using the profile likelihood approach [33]. Since we assume no overdispersion (Poisson distribution), for high read counts (*μ* = 10000) the quadratic approximation of the log-likelihood function applies, and the confidence intervals for the rate estimations may be approximated by the Wald intervals, i.e. $\left(\hat{\delta }-1.96\sqrt{{\left(I^{-1}(\theta )\right)}_{\delta \delta }},\hat{\delta }+1.96\sqrt{{\left(I^{-1}(\theta )\right)}_{\delta \delta }}\right)$, and hence, they reflect the behavior of the FIM term for *δ*. As expected, the relative CI width is minimal only for a certain subset of the rates, depending on the set of measurements included, see (Fig 2B).

If the degradation rate is very fast in comparison to the experiment time scale, the CI width for these fast genes is defined by the earliest time point in the experiment (see Fig 2B).

Since every labeling time is optimal only for a single degradation rate, it might be beneficial to focus the design on genes with faster rates *δ*, if sample size is limited and no other criteria of optimality are given. The justification follows from the faster decay of the FIM term for *α* ≫ 1 (i.e. genes with faster kinetics), Eqs 13 and 14.

### Increasing sample numbers is preferred over higher sequencing depth

Read count data from RNA-seq experiments exhibit overdispersion (variance > mean), and the negative binomial distribution (NB) is the model of choice to account for that [25]. In this section, we explore how overdispersion would affect MLE of *δ*. The overdispersion parameter *k* of the NB distribution describes the level of overdispersion in the data, in which case the variance is defined as var(*X*) = *m* + *m*^2^/*k* for counts *X* ∼ *NB*(*m*, *k*) with mean *m*. Smaller values of *k* correspond to higher overdispersion level, and, for *k* → ∞, the NB distribution converges to the Poisson distribution, for which var(*X*) = *m*. For simplicity, we assume that distributions of read counts in all samples share the same value of *k*. In addition, we do not consider uncertainty in the overdispersion parameter *k* when we make inference about *δ* for individual genes, in a way as it is implemented in some packages for differential expression analysis, for example, in DESeq, [25]. A more advanced quasi-likelihood approach, which accounts for uncertainty in the overdispersion parameter, is discussed in [34].

In the case of NB distribution, the FIM is not diagonal for the SLAMseq experiment, see Eqs 29 and 30 in section 3 of the Extended Methods. Hence we need to work with the inverse FIM, and the diagonal term for the SLAMseq design is

$$\begin{array}{c} {\left(I_{\text{slam}}^{-1}\left(\theta \right)\right)}_{\delta \delta }=\frac{e^{\delta t}-1}{\mu t^{2}}+\frac{2{\left(1-e^{-\delta t}\right)}^{2}}{kt^{2}}. \end{array}$$ (15)

The presence of overdispersion shifts the optimal time to higher values. But the most important change is that the profile of $I^{-1}{\left(\theta \right)}_{\delta \delta }$ is more sensitive to the labeling time *t* near the optimal point. For higher overdispersion values, the variance of the rate estimator $\hat{\delta }$ increases faster in the vicinity of the optimum (see Fig 2C). This imposes stricter conditions on the experimental design. The second term in the Eq 15 vanishes for times *t* ≫ 1, and the equation coincides with the case of no overdispersion. The contribution of the second term is higher for smaller values of *k* (higher overdispersion) and for shorter labeling times *t*, with the maximal value at *t* → 0:

$$\begin{array}{c} \lim_{t\to 0}\frac{2{\left(1-e^{-\delta t}\right)}^{2}}{kt^{2}}=\frac{2\delta^{2}}{k}. \end{array}$$ (16)

Another limitation, which arises in the over-dispersed model is that an increase of the sequencing depth has a limited effect on the variance. Indeed, only the first term in Eq 15 can be eliminated by an increase of sequencing depth:

$$\begin{array}{c} \lim_{\mu \to \infty }{\left(I_{\text{slam}}^{-1}\left(\theta \right)\right)}_{\delta \delta }=\frac{2{\left(1-e^{-\delta t}\right)}^{2}}{kt^{2}}. \end{array}$$ (17)

In contrast, repeating the experiment *n* times affects both terms in $I_{\delta \delta }^{-1}\left(\theta \right)$, since for *n* repetitions,

$$\begin{array}{c} I^{-1}\left(\theta \right)=\frac{1}{n}I_{1}^{-1}\left(\theta \right), \end{array}$$ (18)

where $I_{1}^{-1}\left(\theta \right)$ is the inverse FIM for one repetition.

In the Poissonian case, when *k* → ∞ and the second term is absent (see Eq 9), doubling the number of samples or increasing the sequencing depth by two fold results to the same FIM and, consequently, the same approximation of the variance $\text{var}(\hat{\delta })$. Standard deviation of the rate estimate is a linear function of the depth *μ* on the logarithmic scale and is not bounded below (Fig 2D, dashed line). In contrast, due to Eq 17, presence of overdispersion imposes a limit, which can not be overcome by arbitrary high sequencing depth (Fig 2D, solid line with the horizontal asymptote).

In essence, spreading the sequencing capacity between several biological replicates can be more beneficial than increasing the sequencing depth on a smaller number of samples. A similar phenomenon is discussed by [35] in the context of differential gene expression analysis by RNA-seq.

### Biochemical separation still matters

If one is interested in estimating the rates of extreme values by using very short (e.g. TT-seq, [36]) or long labeling times, it may be less efficient to use the protocols, which preserve the ratio of labeled and unlabeled molecules (e.g. SLAMseq). Let us consider a study of fast gene kinetics, where very short labeling times are used. In this case, *δt* ≪ 1 for the majority of the genes, the labeled fraction constitutes only a minor proportion of the input SLAMseq sample, because *m*_L_(*t*) = *μ*(1 − *e*^−*δt*^) ≈ *μδt* ≪ 1. After a short labeling time, any SLAMseq sample mainly consists of unlabeled molecules from genes with slower synthesis, which leads to spending sequencing resources on mostly non-informative material. The same idea holds for very long times, when *δt* ≫ 1 and when most of the unlabeled molecules were already degraded, *m*_U_(*t*) = *μe*^−*δt*^ ≪ 1.

In contrast, conventional experimental setups with a separation step can be used to focus sequencing capacity on the relevant molecules. However, the conventional approach suffers from the need to normalize sequencing results from different fractions as it does not preserve the ratio of labeled and unlabeled molecules as defined by the input sample. In typical RNA-seq experiments, the normalization coefficients are assumed to be shared between all the genes in a given sample [25], but nevertheless, it introduces additional uncertainty into rate estimations. As previously mentioned, a whole range of normalization approaches has been discussed in literature [26]. In the following derivations, we neglect the uncertainty in estimating the fraction normalization coefficients *x*_*i*_ from Eq 2.

To illustrate the benefit of the conventional approach, let us consider a set of fast turned over genes F, such that there exists labeling time *t*, when the majority of genes i∉F do not contribute to the labeled fractions, i.e. *μ*(1 − *e*^−*δ*_*i*_*t*^) ≪ 1 for i∉F, but *μ*(1 − *e*^−*δ*_*i*_*t*^) ≈ 1 for i∈F. If the sequencing depth of the labeled fraction is approximately the same as for the total sample, then the normalization factor is

$$\begin{array}{c} x_{\text{L}}=\frac{\sum_{i}\mu_{i}}{\sum_{i}\mu_{i}(1-e^{-\delta_{i}t})}\approx \frac{\sum_{i}\mu_{i}}{\sum_{i\in F}\mu_{i}}, \end{array}$$ (19)

which can be high at short times. Such “zooming” effect can be considered as corresponding increase of the sequencing depth in SLAMseq experiments by the factor of *x*_L_ for the labeled fraction. The same idea can be applied to the unlabeled fraction and long labeling times, when the sequencing depth is shared out between the most stable set of genes. Since the normalization factor depends on the rate distribution and the expression level in a given system, it is not possible to derive the optimal design criteria analytically without imposing additional assumptions.

As in the case of SLAMseq, inference can be improved to a limited extent by increase of sequencing depth, if overdispersion is present in the data, compare to Eq 17:

$$\begin{array}{c} \begin{array}{cc} \lim_{\mu \to \infty }{\left(I_{\text{L}}\left(\theta \right)\right)}_{\delta \delta } & =\frac{t^{2}e^{-2\delta t}k}{{\left(1-e^{-\delta t}\right)}^{2}}⩽\frac{k}{\delta^{2}}\\ \lim_{\mu \to \infty }{\left(I_{\text{U}}\left(\theta \right)\right)}_{\delta \delta } & =t^{2}k \end{array} \end{array}$$ (20)

For derivations, see Eqs 58 and 59 in section 4 of Extended Methods. It is interesting to note, that for the case of the unlabeled fraction, the bound can be improved by use of longer labeling times (provided very high sequencing depth), which is not the case for the labeled fraction (with the upper bound $I_{\text{L}}\left(\theta \right)\to k/\delta^{2}$ at *t* → 0).

In summary, biochemical separation should be considered for estimation of degradation rates of RNA species with extreme values. Another design choice is to reduce the number of sequencing reactions by using external spike-ins. For slowly turned over RNA species, one may sequence total and unlabeled fractions, and, for fast turned over RNA species, the total and the labeled fractions. The use of external spike-ins ensures identifiability of the normalizing coefficient from only two fractions.

### Application to a pulse-chase SLAMseq experiment

In this section, we consider a published SLAMseq pulse-chase experiment from [12]. Here, mESCs were treated for 24 hrs with 100 *μ*M 4sU (pulse phase) with samples being collected after 0, 0.5, 1, 3, 6, 12 and 24 hr of label chase, and subjected to QuantSeq mRNA 3’ end sequencing.

While inspecting the data, we noticed that not all the molecules were fully labeled (i.e. not all reads show *T* → *C* conversions) after a 24hr pulse phase. In this case, the labeled fraction does not reach the total level *μ*. We adapted our pulse-chase model to reflect this by introducing a parameter describing the background level of the unlabeled fraction *μ*_1_ and the maximal level of the labeled fraction *μ*_2_, so *μ*_1_ + *μ*_2_ = *μ*:

$$\begin{array}{c} \begin{array}{cc} m_{\text{U}}\left(t\right) & =\mu_{1}+\mu_{2}\left(1-e^{-\delta t}\right)\\ m_{\text{L}}\left(t\right) & =\mu_{2}e^{-\delta t}. \end{array} \end{array}$$ (21)

The equations for the pulse-only experiment and derivations of other results from this section are described in section 3.3 of the Extended methods.

Inefficient nucleotide conversion or too short pulse times may result in high values for the background level *μ*_1_ ≫ *μ*_2_. In this case, the changes due to RNA kinetics, which are proportional to *μ*_2_, constitute only a small part of the read counts, Eq 21. In the extreme case of *μ*_1_/*μ*_2_ → ∞, the unlabeled fraction does not contribute to the FIM term, $\lim_{\mu_{1}/\mu_{2}\to \infty }{\left(I_{\text{U}}\left(\theta \right)\right)}_{\delta \delta }=0$, since it provides information solely on the nuisance parameter for the background *μ*_1_, see Eqs 45 and 47 in Extended Methods. Moreover, if the sequencing depth is fixed to *μ* = *μ*_1_ + *μ*_2_, the amount of labeled molecules is small, *μ*_2_ → 0 as *μ*_1_/*μ*_2_ → ∞ and, hence, ${\left(I_{\text{L}}\left(\theta \right)\right)}_{\delta \delta }\to 0$, see Eq 46 in Extended Methods. It results in high variance of the rate estimate $\hat{\delta }$, because $\text{var}\left(\hat{\delta }\right)={\left(I_{\text{slam}}^{-1}\left(\theta \right)\right)}_{\delta \delta }⩾1/{\left(I_{\text{slam}}\left(\theta \right)\right)}_{\delta \delta }$, but ${\left(I_{\text{slam}}\left(\theta \right)\right)}_{\delta \delta }\to 0$. Consequently, the sequencing capacity is spent for measuring the background level.

Using the inverse FIM to approximate $\text{var}\left(\hat{\delta }\right)={\left(I^{-1}\left(\theta \right)\right)}_{\delta \delta }$ would result in a rather cumbersome expression. But even with our simplified approach, it is possible to see, how inefficient conversion may be detrimental for estimation of *δ* and no design optimization with respect to time of chase-phase could recover the situation.

To illustrate, how the choice of time point affects the confidence intervals of the estimations, we analyzed different subsets of samples from [12]. Since the model includes one more parameter to take the background level into account (*μ*_1_), one needs to use at least two different time points. In our example, we use combinations of different chase-times and always include *t* = 0, because these samples directly provide the information on the *μ*_1_ and *μ*_2_, since *m*_U_(0) = *μ*_1_ and *m*_L_(0) = *μ*_2_.

**Fig 3:**
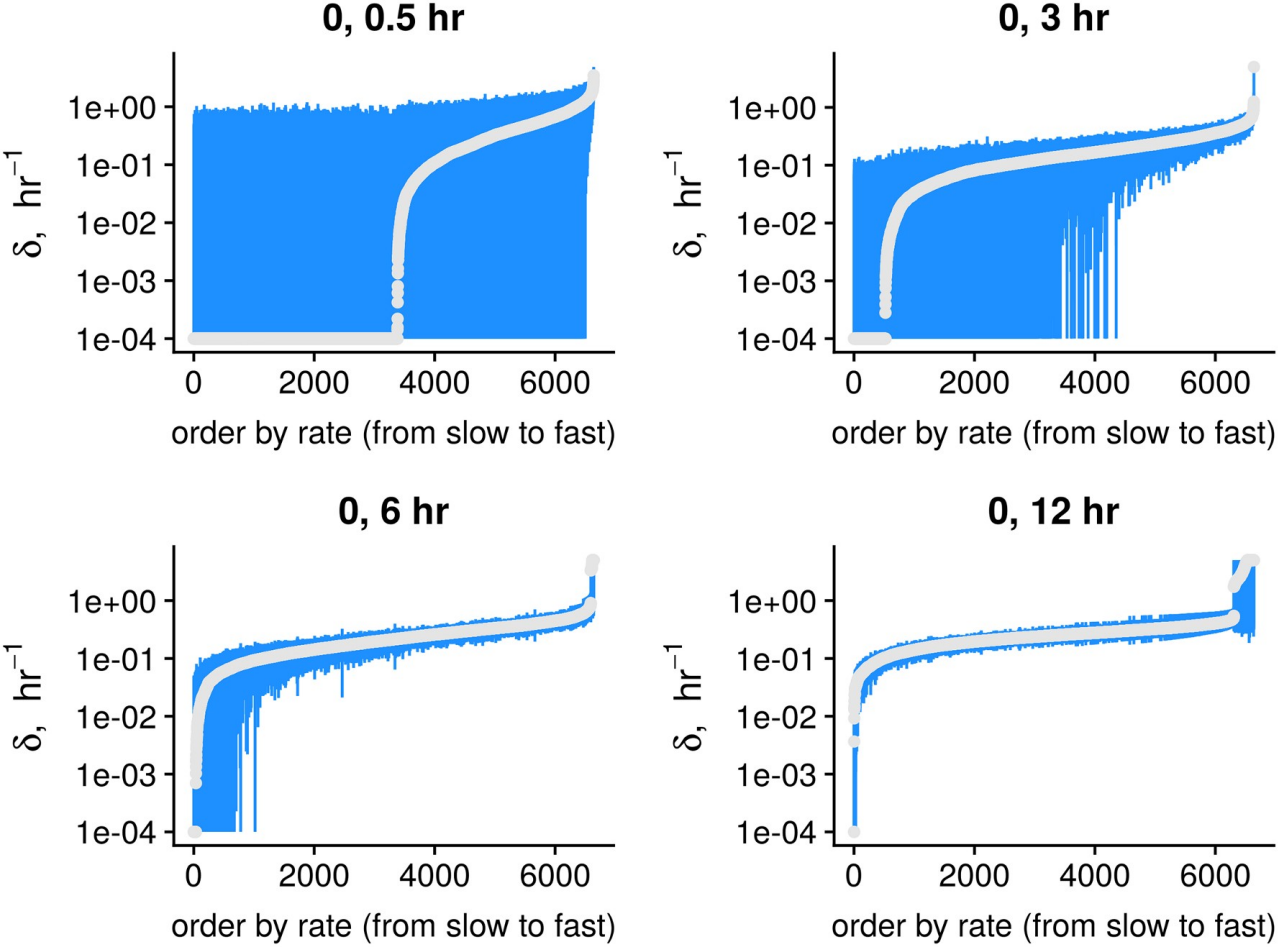
Estimates for pulse-chase SLAM-seq data [12]. Degradation rates and 95% confidence intervals are shown for different chase time points. For short chase times, the majority of genes have poorly identified degradation rates (see subsets [0, 0.5], [0, 3], [0, 6] hr). On the other hand, longer chase times do not allow to precisely estimate rates for unstable genes ([0, 12] hr).

As expected, for a short (relative to the characteristic time) chase phase, subtle changes in the levels of the labeled and unlabeled molecules are masked by the noise and the majority of the degradation rates are not identifiable (Fig 3, sample sets for [0, 0.5] hr, [0, 3] hr). Using one more early time point ([0, 0.5, 1] hr) did not substantially improve the estimates, S1 Fig. At longer chase phases, the confidence intervals are more narrow ([0, 6] hr), and for longer time the estimations for fast genes become worse, since most of their labeled RNA molecules are already degraded ([0, 12] hr, right side of the x-axis).

To illustrate, how the FIM term for a single sample depends on the time of the chase phase, we calculated ${\left(I_{\text{slam}}\left(\theta \right)\right)}_{\delta \delta }{\hat{\delta }}^{2}$ for a range of different values of *t*/*τ* ratio. ${\left(I_{\text{slam}}\left(\theta \right)\right)}_{\delta \delta }{\hat{\delta }}^{2}$ depends on other parameters as well (see Eq. 48 in section 3.3 of the Extended Methods). In this example, we used parameter values from the model fitted to the full data set, i.e. including 0, 0.5, 1, 3, 6, 12 and 24 hr chase time points (overdispersion parameter $\hat{k}=10.4$ and medians of ${\hat{\mu }}_{1}$ and ${\hat{\mu }}_{2}$, 251 and 89 correspondingly).

**Fig 4:**
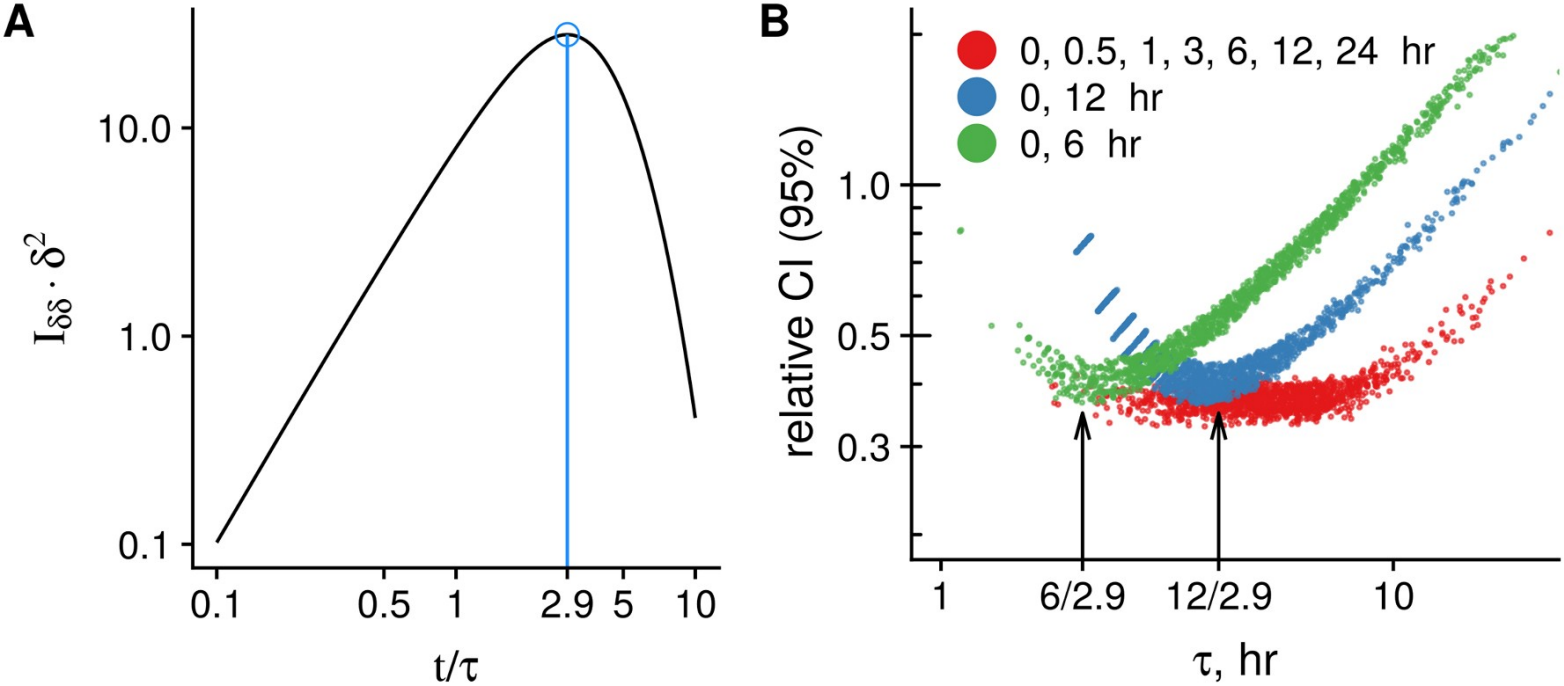
Application to the SLAMseq experiment. **A**: Diagonal term of the FIM ${\left(I\left(\theta \right)\right)}_{\delta \delta }$ as a function of chase time. Similar to Fig 2, we normalize it as ${\left(I\left(\theta \right)\right)}_{\delta \delta }\delta^{2}$, so it corresponds to the lower boundary of the relative variance $\text{var}\left(\hat{\delta }\right)/\delta^{2}⩾1/\left({\left(I\left(\theta \right)\right)}_{\delta \delta }\delta^{2}\right)$. Using time points with low ${\left(I\left(\theta \right)\right)}_{\delta \delta }$ values results in higher variance of $\hat{\delta }$. In this example, as values of ${\hat{\mu }}_{1}$ and ${\hat{\mu }}_{2}$, we use medians of their estimations from the model fitted to the full set of points. **B**: Relative width of 95% confidence intervals (CI) for the rate estimations $\hat{\delta }$. We use the genes with ${\hat{\mu }}_{2}$ located between 40%-60% percentiles (i.e. near the median). Genes, which have ratio close to the optimum *t*/*τ* ≈ 2.9 (subfigure (A)), have smaller relative CI for $\hat{\delta }$.

Similar to the simpler case in Fig 2A, there is an optimal time, where ${\left(I_{\text{slam}}\left(\theta \right)\right)}_{\delta \delta }{\hat{\delta }}^{2}$ is maximal, *t* ≈ 2.9*τ* (Fig 4A). Genes with a characteristic time *τ*, which diverge from *t*/2.9, will have confidence intervals with a large relative width, and, *vice versa*, the relative interval width will be more narrow for the genes with *τ* ≈ *t*/2.9.

To be in line with our estimation for a gene with the median ${\hat{\mu }}_{2}$, we plotted the genes with ${\hat{\mu }}_{2}$ located around the median (in 40-60% percentile) for illustration. For 6 and 12 hr points, there is a distinct minimum in relative confidence intervals at 6/2.9 ≈ 2 hr and 12/2.9 ≈ 4 hr (Fig 4B). The median of the characteristic time estimates is $\hat{\tau }=5.4$ hr, and the optimal chase time for such “median” gene would be around 15 hr. In agreement with this observation, the degradation rate estimates, calculated using [0, 12] hr and [0, 24] hr points, have the highest correlation to the rates, which were derived from the full data set (S2 Fig).

Although in majority of cases several different time points are used, the results of this section show that too long or too short times barely contribute to the estimations. Another factor, which influences the quality, is efficiency of the labeling protocol. The presence of non-informative background RNA creates additional noise to the measurements and wastes sequencing capacity.

### Example from a pulse labeling experiment

**Fig 5:**
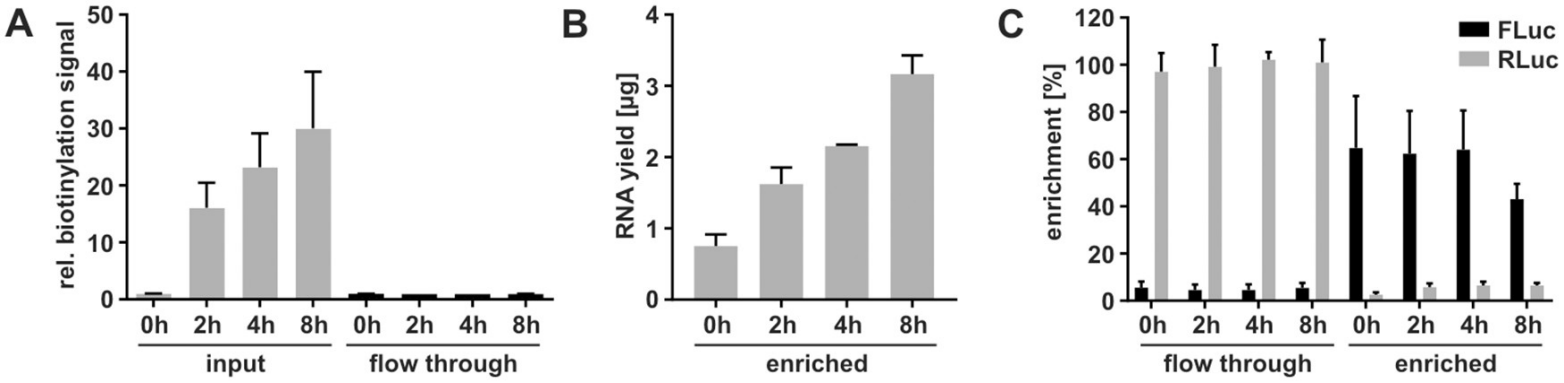
Purification of labeled and unlabeled RNA fractions. MCF-7 cells were pulse labeled with 4sU for up to eight hr as indicated. Total RNA was spiked with *in vitro* transcribed 4sU-labeled FLuc and unlabeled RLuc, biotinylated with MTSEA-biotin and subjected to streptavidin purification. (n = 3). **A**: Dot blot-based detection of biotinylation with streptavidin-HRP in input and flow through of streptavidin purification. **B**: The amount of RNA enriched by the streptavidin purification was determined by absorption measurement. **C**: *In vitro* transcribed spike in RNAs 4sU-labeled FLuc and unlabeled RLuc in the flow through and biotin-enriched fraction were measured by RT-qPCR analysis and normalized to a standard curve given in S3 Fig.

MCF-7 cells were pulse labeled with 200 *μ*M 4sU for 2, 4 or 8 hrs. 4sU-labeled and unlabeled RNA were separated by streptavidin purification after MTSEA biotin-XX catalyzed biotinylation of 4sU-labeled RNA, which has an efficiency of 95% [18]. The efficiency of purification was monitored in a dot blot assay that detects biotinylated RNA with streptavidin-HRP (Fig 5A, S3 Fig). This analysis revealed a gradual increase in biotinylation with increasing labeling time. Importantly, biotinylated transcripts were efficiently depleted from the flow through. No biotinylation signal could be detected in these samples, which illustrates the high efficiency of the streptavidin purification. Biotin-enriched RNAs are eluted by three rounds of de-biotinylation with DTT. Therefore, we estimated the purification efficiency by the amount of purified RNA determined by A_260nm_ absorption measurement. The amount of purified RNA increased gradually with increasing labeling time (Fig 5B) comparable to the biotinylation signal increase in the respective input fractions (Fig 5A). To determine the efficiency and specificity precisely for individual transcripts, we spiked the 4sU-labeled total RNA from MCF-7 with *in vitro* transcribed 4sU-labeled FLuc and unlabeled RLuc that were followed by RT-qPCR analysis using a standard curve for quantification (S3 Fig). This analysis revealed a purification efficiency of 4sU-labeled FLuc of about 60% (58.56). The specificity was determined by the cross-contamination of RLuc in the biotin-enriched fractions and FLuc in the flow through fractions, which was about 5% for each transcript (RLuc in enriched = 5.32%, FLuc in flow through = 5.01%, see Fig 5C).

The kinetic model was fitted to the read counts from the sequenced samples for genes with mean read count >50 in the total samples. Two total samples were collected at 0 hr, labeled and unlabeled fractions at other time points (2, 4 and 8 hrs) in two replicates (see S2 Table). In the model fitting, we assumed no cross-contamination between fractions and shared normalization coefficients for samples originating from the same time point and fraction.

Having the estimations for expression levels, degradation rates, overdispersion parameter and normalization coefficients, we calculated the FIM diagonal elements $I_{\delta \delta }\left(\theta \right)$ for the analyzed genes for different time points and fraction types.

**Fig 6:**
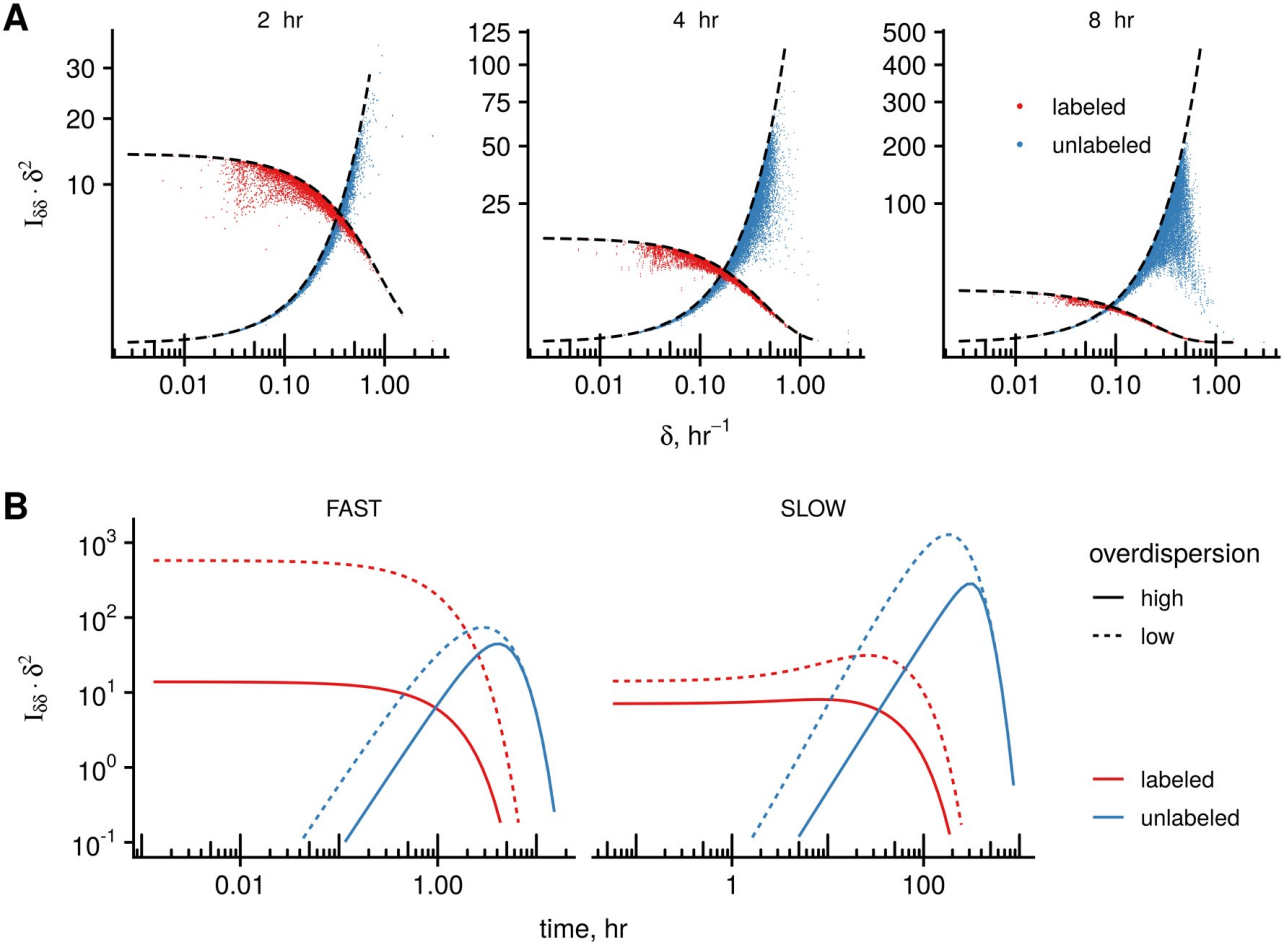
Application to experimental data from the MCF-7 pulse labeling time course experiment. **A**: We plot the diagonal term of the FIM computated at estimated parameter values and multiplied by ${\hat{\delta }}^{2}$, $I_{\delta \delta }\left(\theta \right){\hat{\delta }}^{2}$, to illustrate contributions from labeled and unlabeled fractions to estimations of degradation rates for different experimental points (MCF-7 experiment, 2, 4, and 8 hr) and fractions (labeled and unlabeled). The black lines are the limiting values for the $I_{\delta \delta }\left(\theta \right)$ according to Eq 20. **B**: The modified FIM term $I_{\delta \delta }\left(\theta \right){\hat{\delta }}^{2}$ is computed for a range of labeling times for one of the fastest (at the 0.1% quantile) and one of the slowest (at the 99,9% quantile) genes (*δ*_fast_ = 0.79hr^−1^, *δ*_slow_ = 0.019hr^−1^). The normalization coefficient for the labeled and unlabeled fractions is adjusted in such a way that their sequencing depth (total mean read count) at time *t* equals the sequencing depth of the total sample.

In Fig 6A, the value of the diagonal FIM element multiplied by ${\hat{\delta }}^{2}$, i.e. $I_{\delta \delta }\left(\theta \right){\hat{\delta }}^{2}$ (compare to Eqs 11 and 12), is depicted for both fractions. As mentioned in the previous section, $I_{\delta \delta }\left(\theta \right)$ can be interpreted as an information gain from the experiment assuming other parameters were known, which represents an upper bound, see Eq 8. In addition, these terms are bounded due to presence of overdispersion in the data, (Eq 20 and dashed lines in Fig 6A), and increase of sequencing depth can not improve these limits.

At short labeling times, the FIM term is higher for the labeled fraction than for the unlabeled one for majority of the genes, (Fig 6A, 2hr), which is a result similar to the SLAMseq case. At longer labeling times, the contribution from the unlabeled fraction increases, and ${\left(I_{\text{U}}(\theta )\right)}_{\delta \delta }>{\left(I_{\text{L}}(\theta )\right)}_{\delta \delta }$ for majority of the genes (Fig 6A, 8hr). However, the proportion of RNA amount from genes with high degradation rates *δ* in the unlabeled sample exponentially decreases, since

$$\begin{array}{c} \lim_{t\to \infty }\frac{\mu_{\text{fast}}e^{-\delta_{\text{fast}}t}}{\mu_{\text{slow}}e^{-\delta_{\text{slow}}t}}=\lim_{t\to \infty }\frac{\mu_{\text{fast}}}{\mu_{\text{slow}}}e^{-(\delta_{\text{fast}}-\delta_{\text{slow}})t}=0. \end{array}$$ (22)

It results in very low counts and decrease in the $I_{\text{U}}\left(\theta \right)$ for these fast genes, see Fig 6A, 8hr, reduced values at the right tail of the distribution (blue dots).

The optimal design for such experiments is complicated by the fact that it depends not only on the degradation rates of some target genes, but on the overall rate distribution in the system being studied. We illustrate a dependency of the ${\left(I(\theta )\right)}_{\delta \delta }\delta^{2}$ terms on labeling time for one of the fastest (0.1% quantile) and one of the slowest (99.9% quantile) genes. The normalization coefficients for the labeled and unlabeled fractions were adjusted in such a way, that at every time point *t* the sequencing depth equals the sequencing depth of the total sample. In the case of low or no overdispersion, use of labeled fraction and shorter labeling times is preferred for estimation of fast genes, because ${\left(I_{\text{L}}\left(\theta \right)\right)}_{\delta \delta }>{\left(I_{\text{U}}\left(\theta \right)\right)}_{\delta \delta }$, see Fig 6B, dashed red line over the blue line. For slow genes, one may benefit from use of unlabeled fraction, since the highest FIM values correspond to ${\left(I_{\text{U}}\left(\theta \right)\right)}_{\delta \delta }$ at longer times, see Fig 6B, dashed blue line over red line.

At high values of overdispersion (i.e. low *k*), the FIM term is bounded ${\left(I_{\text{L}}\left(\theta \right)\right)}_{\delta \delta }\delta^{2}<k$ due to Eq 20. In this case, there may exist values of labeling times at which the terms from the unlabeled fraction ${\left(I_{\text{U}}\left(\theta \right)\right)}_{\delta \delta }\delta^{2}$ is larger than maximal ${\left(I_{\text{L}}\left(\theta \right)\right)}_{\delta \delta }\delta^{2}$ value, Fig 6B, solid lines. As a protection against such situation in the case of fast genes, use of samples from unlabeled fraction may be a solution. Although one may have a prior guess about the range of degradation rates in a system, it is unlikely that there is information about the distribution of the rates and overdispersion level. Hence, such design suggestions are possible only in sequential approach, when an exploratory experiment is done first.

It is important to note the “zooming” effect of the conventional design, which we discussed in the previous section *Biochemical separation still matters*. At a short labeling time, the term ${\left(I_{\text{L}}\left(\theta \right)\right)}_{\delta \delta }\delta^{2}$ decreases as *t* approches zero in the case of the SLAMseq design, Fig 2A, the red line. In contrast, due to higher sequencing depth of individual fractions in the conventional setting, ${\left(I_{\text{L}}\left(\theta \right)\right)}_{\delta \delta }\delta^{2}$ has a horizontal asymptote, Fig 6B, red lines.

## Discussion

In this study, we discuss some aspects of the optimal design of RNA labeling experiments using the results of the asymptotic theory. First, we show that there exists an optimal time point for which the maximum likelihood estimator possess a minimal variance asymptotically. This first result was developed for the case of experiments, which preserve the fraction ratio and hence do not require normalization between fractions (e.g. SLAMseq, TUC-seq, TimeLapse-seq).

In the case of negligible overdispersion, the optimal labeling time for a gene with the characteristic degradation time *τ* is *t*_slam_ = 1.59*τ*, and shorter labeling times show better rate estimates in comparison to longer times: the variance increases exponentially for times longer than *τ* and only by a power law for shorter labeling times. This result is similar to the observations in a simulation study by [32]. Herein, for a given gene with a half-life λ = 2 hr, the most precise estimation were at labeling times 3 hr and 6 hr (*t*_optimal_ = 1.59 ⋅ 2/log(2) = 4.6 hr), and the worst estimations were observed at the longest and the shortest times (12 hr and 0.5 hr). However, the exact ranking of time points is different for the given half-life time, probably due to the influence of prior distribution utilized in the Bayesian framework.

We show that at short labeling times (in comparison to the characteristic time of degradation for a given gene), the labeled fraction contributes most to the Fisher information term corresponding to the degradation rate, and, *vice versa*, at long times the highest contribution is seen for the unlabeled one.

In addition, we show that in the presence of overdispersion, the variance of rate estimates is more sensitive to choices of labeling times different from the optimal, which make it more difficult to optimize conditions for a range of rates. The overdispersion imposes a bound on the asymptotic relative standard deviation for the estimator of the rate (*sd*(*δ*)/*δ*, see Fig 2C), and, from a certain level, increase in sequencing depth is very inefficient (Fig 2D).

We present similar results for SLAMseq data from a published pulse-chase experiment. Herein, we extended our model to reflect incomplete labeling and demonstrate that every chase time is optimal only for genes with a certain ratio of the characteristic degradation time and the chase time (*t*_slam_ ≈ 2.9*τ*, see Fig 4).

Moreover, we discuss possible benefits of use of the conventional experimental approach, especially for estimation of extreme degradation rates, which deviate highly from the general pool. For nucleotide conversion setups with too short or too long labeling times, the majority of reads in a sample originate from the unlabeled or labeled fractions correspondingly. In contrast, the conventional scheme, which involves biochemical fraction separation, allows to concentrate the experimental costs only on the relevant material. This approach strongly relies on normalization between the samples, as the fraction ratio is not preserved. Besides the use of labeled and unlabeled spike ins additional normalization strategies have been developed to ensure this, see [26].

Obviously, there are certain limitations to our study. First, the method involving FIM calculation describes only the asymptotic behavior of the estimator. Hence, all the conclusions are only approximate, since we do not investigate the behavior of the likelihood function itself, but only the quadratic approximation of its logarithm using the FIM.

Secondly, we do not consider uncertainty from the shared parameters, such as the overdispersion parameter of the negative binomial distribution and the normalization coefficients for the fractions. Inference on these parameters is based on the whole pool of the genes, and would involve more complex analytic treatment and assumptions on the distribution of rates.

Thirdly, this study is concerned with the statistical aspects, rather than kinetic modeling, and the simplest model of synthesis and degradation is used. More complex models, which describe biochemical networks or RNA maturation can be more relevant depending on the research question. Other phenomena, like dilution due to cell division, may have an effect on the RNA level as well and should be taken into account in the case of the long-lived transcripts [26].

Lastly, cross-contamination between fractions is a highly relevant problem for inference, especially in the absence of external reference molecules (spike ins), which are typically used to assess this phenomenon. However, in section 2.1 of the Extended methods, we show that cross-contamination shifts estimations of fast rates to slower values, and slow rates towards faster values. Previously, [28] included a global transcriptome-wide cross-contamination term to presented kinetic model, yet future work is needed to assess possible effect sizes on rate estimations.

With regards to our own experimental results, we used unlabeled RLuc and 4sU-labeled FLuc to control the efficiency and specificity of biochemical separation. We reckon that the recovery of only 65% 4sU-labeled FLuc may be caused by inefficient elution or loss during the washing steps. RNA species with a high 4sU content are more likely to be affected by inefficient elution, whereas the loss during the washing steps may be observed for RNAs with very few 4sU. These effects will also introduce a bias in rate estimates, which originate from the biotin-enriched fraction.

We hope that our work will encourage further development of the methodology to address the discussed limitations and to improve suggestions on design of metabolic labeling experiments.

## Supporting information

S1 File Extended methods. Supplementary PDF document with additional mathematical derivations and details.
(PDF)

S1 FigProfile likelihood confidence intervals.95% profile likelihood confidence intervals for the estimates of the degradation rate $\hat{\delta }$, derived from the time points at 0, 0.5 and 1 hr of the chase phase.(TIFF)

S2 Fig Spearman correlation between estimates of degradation rates. Spearman correlation between estimates of degradation rates, computed for different subsets of time points, and the rates derived from the whole data set.
(TIFF)

S3 Fig Assessment of biotinylation status and standard curves for spike in quantification. **A**: Dot blot-based detection of biotinylation with streptavidin-HRP in input and flow through of streptavidin purification from three replicate experiments A-C. The quantification of the captured image is shown in Fig 3A. **B**: Standard curve for the absolute quantification of 4sU-labeled FLuc RNA. 1600 to 1.56% of the input used for streptavidin purification was measured by RT-qPCR analysis in 1:2 dilutions. The log10 amount of RNA was plotted against the obtained Ct value and used for linear regression. **C**: Standard curve for the absolute quantification of unlabeled RLuc RNA. 400 to 3.13% of the input used for streptavidin purification was measured by RT-qPCR analysis in 1:2 dilutions. The log10 amount of RNA was plotted against the obtained Ct value and used for linear regression.
(TIFF)

S1 Table Summary of RNA-seq read mapping statistics. (XLSX)

S2 Table Read counts for all samples. https://github.com/dieterich-lab/DesignMetabolicRNAlabeling.
(XLSX)

## References

[1] Schwanhäusser B, Busse D, Li N, Dittmar G, Schuchhardt J, Wolf J, et al Global quantification of mammalian gene expression control. Nature. 2011;473:337–342. 10.1038/nature10098 21593866

[2] Ziaeian B, Fonarow GC. Epidemiology and aetiology of heart failure. Nature reviews Cardiology. 2016;13:368–378. 10.1038/nrcardio.2016.25 26935038 PMC4868779

[3] Wilson WR, Hay MP. Targeting hypoxia in cancer therapy. Nature reviews Cancer. 2011;11:393–410. 10.1038/nrc3064 21606941

[4] Johnson AB, Denko N, Barton MC. Hypoxia induces a novel signature of chromatin modifications and global repression of transcription. Mutation research. 2008;640:174–179. 10.1016/j.mrfmmm.2008.01.001 18294659 PMC2346607

[5] Semenza GL. Targeting HIF-1 for cancer therapy. Nature reviews Cancer. 2003;3:721–732. 10.1038/nrc1187 13130303

[6] Huang LE, Gu J, Schau M, Bunn HF. Regulation of hypoxia-inducible factor 1alpha is mediated by an O2-dependent degradation domain via the ubiquitin-proteasome pathway. Proceedings of the National Academy of Sciences of the United States of America. 1998;95:7987–7992. 10.1073/pnas.95.14.7987 9653127 PMC20916

[7] Gorospe M, Tominaga K, Wu X, Fähling M, Ivan M. Post-Transcriptional Control of the Hypoxic Response by RNA-Binding Proteins and MicroRNAs. Frontiers in Molecular Neuroscience. 2011;4:7 10.3389/fnmol.2011.00007 21747757 PMC3130151

[8] Cleary MD, Meiering CD, Jan E, Guymon R, Boothroyd JC. Biosynthetic labeling of RNA with uracil phosphoribosyltransferase allows cell-specific microarray analysis of mRNA synthesis and decay. Nature Biotechnology. 2005;23:232–237. 10.1038/nbt1061 15685165

[9] Dölken L, Ruzsics Z, Rädle B, Friedel CC, Zimmer R, Mages J, et al High-resolution gene expression profiling for simultaneous kinetic parameter analysis of RNA synthesis and decay. RNA. 2008;14(9):1959–1972. 10.1261/rna.1136108 18658122 PMC2525961

[10] Wachutka L, Gagneur J. Measures of RNA metabolism rates: Toward a definition at the level of single bonds. Transcription. 2017;8:75–80. 10.1080/21541264.2016.1257972 27841720 PMC5423486

[11] Baptista MAP, Dölken L. RNA dynamics revealed by metabolic RNA labeling and biochemical nucleoside conversions. Nature Methods. 2018;15:171–172. 10.1038/nmeth.4608 29489745

[12] Herzog VA, Reichholf B, Neumann T, Rescheneder P, Bhat P, Burkard TR, et al Thiol-linked alkylation of RNA to assess expression dynamics. Nature Methods. 2017;14:1198–1204. 10.1038/nmeth.4435 28945705 PMC5712218

[13] Schofield JA, Duffy EE, Kiefer L, Sullivan MC, Simon MD. TimeLapse-seq: adding a temporal dimension to RNA sequencing through nucleoside recoding. Nature Methods. 2018;15:221–225. 10.1038/nmeth.4582 29355846 PMC5831505

[14] Riml C, Amort T, Rieder D, Gasser C, Lusser A, Micura R. Osmium-Mediated Transformation of 4-Thiouridine to Cytidine as Key To Study RNA Dynamics by Sequencing. Angewandte Chemie (International ed in English). 2017;56:13479–13483. 10.1002/anie.201707465 28817234

[15] Russo J, Heck AM, Wilusz J, Wilusz CJ. Metabolic labeling and recovery of nascent RNA to accurately quantify mRNA stability. Methods. 2017;120:39–48. 10.1016/j.ymeth.2017.02.003 28219744 PMC5447484

[16] Liepelt A, Mossanen JC, Denecke B, Heymann F, De Santis R, Tacke F, et al Translation control of TAK1 mRNA by hnRNP K modulates LPS-induced macrophage activation. RNA. 2014;. 10.1261/rna.042788.113 24751651 PMC4024643

[17] Thermann R, Hentze MW. Drosophila miR2 induces pseudo-polysomes and inhibits translation initiation. Nature. 2007;447(7146):875 10.1038/nature05878 17507927

[18] Duffy EE, Rutenberg-Schoenberg M, Stark CD, Kitchen RR, Gerstein MB, Simon MD. Tracking Distinct RNA Populations Using Efficient and Reversible Covalent Chemistry. Molecular Cell. 2015;59:858–866. 10.1016/j.molcel.2015.07.023 26340425 PMC4560836

[19] de Vries S, Naarmann-de Vries IS, Urlaub H, Lue H, Bernhagen J, Ostareck DH, et al Identification of DDX6 as a cellular modulator of VEGF expression under hypoxia. Journal of Biological Chemistry. 2013; p. jbc–M112. 10.1074/jbc.M112.420711 PMC3581395 23293030

[20] Naarmann-de Vries IS, Brendle A, Bähr-Ivacevic T, Benes V, Ostareck DH, Ostareck-Lederer A. HnRNP K-mediated translational control links NMHC IIA to erythroid enucleation. J Cell Sci. 2016; p. jcs–174995. 10.1242/jcs.174995 26823606

[21] Roehr JT, Dieterich C, Reinert K. Flexbar 3.0—SIMD and multicore parallelization. Bioinformatics (Oxford, England). 2017;33:2941–2942. 10.1093/bioinformatics/btx330 28541403

[22] Langmead B, Salzberg SL. Fast gapped-read alignment with Bowtie 2. Nature methods. 2012;9:357–359. 10.1038/nmeth.1923 22388286 PMC3322381

[23] Dobin A, Davis CA, Schlesinger F, Drenkow J, Zaleski C, Jha S, et al STAR: ultrafast universal RNA-seq aligner. Bioinformatics (Oxford, England). 2013;29:15–21. 10.1093/bioinformatics/bts635 PMC3530905 23104886

[24] Pertea M, Pertea GM, Antonescu CM, Chang TC, Mendell JT, Salzberg SL. StringTie enables improved reconstruction of a transcriptome from RNA-seq reads. Nature Biotechnology. 2015;33:290–295. 10.1038/nbt.3122 25690850 PMC4643835

[25] Anders S, Huber W. Differential expression of RNA-Seq data at the gene level–the DESeq package. Heidelberg, Germany: European Molecular Biology Laboratory (EMBL) 2012;.

[26] Lugowski A, Nicholson B, Rissland OS. Determining mRNA half-lives on a transcriptome-wide scale. Methods. 2018;137:90–98. 10.1016/j.ymeth.2017.12.006 29247756

[27] Miller C, Schwalb B, Maier K, Schulz D, Dümcke S, Zacher B, et al Dynamic transcriptome analysis measures rates of mRNA synthesis and decay in yeast. Molecular Systems Biology. 2011;7(1):458 10.1038/msb.2010.112 21206491 PMC3049410

[28] Eser P, Wachutka L, Maier KC, Demel C, Boroni M, Iyer S, et al Determinants of RNA metabolism in the Schizosaccharomyces pombe genome. Molecular Systems Biology. 2016;12(2):857 10.15252/msb.20156526 26883383 PMC4770384

[29] Chernoff H. Locally optimal designs for estimating parameters. The Annals of Mathematical Statistics. 1953; p. 586–602. 10.1214/aoms/1177728915

[30] Pawitan Y. In all likelihood: statistical modelling and inference using likelihood. Oxford University Press; 2001.

[31] Van den Bos A. Parameter estimation for scientists and engineers. John Wiley & Sons; 2007.

[32] Jürges C, Dölken L, Erhard F. Dissecting newly transcribed and old RNA using GRAND-SLAM. Bioinformatics. 2018;34(13):i218–i226. 10.1093/bioinformatics/bty256 29949974 PMC6037110

[33] Uvarovskii A, Dieterich C. pulseR: Versatile computational analysis of RNA turnover from metabolic labeling experiments. Bioinformatics. 2017;33(20):3305–3307. 10.1093/bioinformatics/btx368 29028260

[34] Lund SP, Nettleton D, McCarthy DJ, Smyth GK. Detecting differential expression in RNA-sequence data using quasi-likelihood with shrunken dispersion estimates. Statistical applications in Genetics and Molecular Biology. 2012;11(5). 10.1515/1544-6115.1826 23104842

[35] Robles JA, Qureshi SE, Stephen SJ, Wilson SR, Burden CJ, Taylor JM. Efficient experimental design and analysis strategies for the detection of differential expression using RNA-Sequencing. BMC Genomics. 2012;13(1):484 10.1186/1471-2164-13-484 22985019 PMC3560154

[36] Schwalb B, Michel M, Zacher B, Frühauf K, Demel C, Tresch A, et al TT-seq maps the human transient transcriptome. Science. 2016;352(6290):1225–1228. 10.1126/science.aad9841 27257258

