# Supplementary material for: On the optimal design of metabolic RNA labeling experiments
Source: PLoS Comput Biol. 2019 Aug 7;15(8):e1007252. doi: 10.1371/journal.pcbi.1007252 (PMC6699717; doi:10.1371/journal.pcbi.1007252)
Supplement: S1 File — Supplementary PDF document with additional mathematical derivations and details. (PDF) [file pcbi.1007252.s001.pdf]

On the optimal design of metabolic RNA labeling experiments

Alexey Uvarovskii[1,2,3] , Isabel S. Naarmann-de Vries[4,¶] and Christoph Dieterich[1,2,¶,#]

1: Section of Bioinformatics and Systems Cardiology, Klaus Tschira Institute for Integrative Computational Cardiology and Department of Internal Medicine III, University Hospital Heidelberg
2: German Center for Cardiovascular Research (DZHK)
3: Current address: Roche Diagnostics GmbH, Nonnenwald 2, 82377 Penzberg
4: Department of Intensive Care Medicine, University Hospital Aachen, RWTH Aachen University
Correspondence to *alexey.mipt@gmail.com and christoph.dieterich@uni-heidelberg.de*
¶: equal contribution #: Lead contact

July 28, 2019

Extended Methods

1 Statistical model

We assume that the read counts follow the negative binomial distribution with the probability distribution function

$$P(X=x)=\frac{\Gamma(k+x)}{x!\Gamma(k)}\left(\frac{m}{m+k}\right)^{x}\left(\frac{m+k}{k}\right)^{-k}, \tag{1}$$

where $m = m(\mu, d, \ldots, t)$ is the mean read count expected from the kinetic model and depends on the time point t, expression level in a steady-state μ, degradation rate δ, and sample normalization, see the next section for details. The negative binomial distribution imposes a relation between variance and mean via the overdispersion parameter k, $\mathrm{var}(X) = m(m + k)/k$. We assume the same k for all the genes in the data set, which is the simplest model, but more complicated models exist [Anders and Huber, 2012]. In this case, k is a shared parameter, and the model parameters from different genes must be fitted together in one procedure.

The logarithm of the likelihood function depends on the experimental points $X_1, \ldots, X_n$ and the vector of all model parameters $\boldsymbol{\theta}$

$$\log \mathcal{L}(\boldsymbol{\theta}, X) = \sum_i \log P(\boldsymbol{\theta}, X_i). \tag{2}$$

The maximum likelihood estimator is then

$$\hat{\boldsymbol{\theta}} = \arg\max_{\boldsymbol{\theta}} \log \mathcal{L}(\boldsymbol{\theta}, X). \tag{3}$$

2 Kinetic model

The solution of the differential equation for the kinetics of synthesis and degradation of the RNA $\frac{dm}{dt} = s - \delta m$ is

$$m(t) = m_0 e^{-\delta t} + (1 - e^{-\delta t}) s/\delta = \mu + (m_0 - \mu) e^{-\delta t}, \tag{4}$$

where m_0 is the initial amount of RNA, $\mu = s/\delta$ is the expression level in the steady state for synthesis rate s and degradation rate δ.

For definiteness, consider a pulse experiment. The unlabeled fraction is being only degraded (synthesis rate $s = 0$), and the initial RNA level is μ

$$m_{\mathrm{U}}(t) = \mu e^{-\delta t}. \tag{5}$$

The labeled fraction starts from zero and saturates to the steady state:

$$m_{\mathrm{L}}(t) = \mu(1 - e^{-\delta t}). \tag{6}$$

In fact, the mean read count is only proportional to the amount of RNA in samples, so without spike-in fragments added to measure absolute concentration, we can estimate the amounts only up to unknown coefficient. For identifiability, we use the read counts in the total samples as a reference (accounting for difference in sequencing depth, as implemented in the `DESeq` package [Anders and Huber, 2012]).

If the ratio of fractions is preserved, as, for example, in the SLAMseq protocol, the counts in the labeled X_{L} and the unlabeled X_{U} fractions are scaled by the same sequencing depth correction x, $\mathbb{E}(X_{\mathrm{U}} + X_{\mathrm{L}}) = x\mu$ and

$$\mathbb{E}X_{\mathrm{U}} = x \cdot \mu e^{-\delta t} \tag{7}$$

$$\mathbb{E}X_{\mathrm{L}} = x \cdot \mu(1 - e^{-\delta t}) \tag{8}$$

Assuming that usually samples are sequenced to approximately the same depth, for theoretical derivations we use $x = 1$ for the SLAMseq experiment.

If the fractions were separated by a chemical procedure, the read counts ratio will not coincide with the ratio of labeled and unlabeled molecules. In the case of negligible cross-contamination,

$$\begin{aligned} \mathbb{E}X_{\mathrm{U}} &= x_1 \cdot \mu e^{-\delta t} \\ \mathbb{E}X_{\mathrm{L}} &= x_2 \cdot \mu(1 - e^{-\delta t}). \end{aligned} \tag{9}$$

2.1 Bias due to cross-contamination

In this work, we do not consider cross-contamination, however it can play a significant role, especially for extreme rates (very fast or very slow) even if the overall contaminated material amount is low.

Indeed, if there is a cross-contamination level of γ, the mean read count can be modeled as

$$\mathbb{E}X_{\mathrm{U}} = x_1 \left((1-\gamma) \cdot \mu e^{-\delta t} + \gamma \cdot \mu \left(1 - e^{-\delta t}\right)\right). \tag{10}$$

Under the model, which does not accommodate for cross-contamination, the degradation rate estimate δ^* is biased:

$$\begin{aligned} \mathbb{E}X_{\mathrm{U}} &= x_1^* \mu^* e^{-\delta^* t} \approx x_1 \mu e^{-\delta^* t} = x_1 \mu \left(e^{-\delta t} + \gamma \left(1 - 2e^{-\delta t}\right)\right) \\ \delta^* &= -\frac{1}{t} \ln \left(e^{-\delta t} + \gamma \left(1 - 2e^{-\delta t}\right)\right), \end{aligned} \tag{11}$$

where we assume, that $x_{\mathrm{i}}^* \approx x_{\mathrm{i}}$, $\mu^* \approx \mu$, in order to simplify our illustration. In general case, however, cross-contamination may affect normalization coefficients x_{i} and the estimates of the expression level μ. Influence of contamination is more pronounced for extreme rates, in which case $e^{-\delta t} \approx 1$ or $e^{-\delta t} \approx 0$, and it has minimal effect if $e^{-\delta t} \approx 1/2$. Let us consider such small values of γ, that, for majority of genes, estimates are negligibly affected. If x_{i} are derived from the whole pool of genes, we can assume $x_1^* \approx x_1$.

These derivations are relevant to the SLAMseq method as well, where misclassification of the reads has the same effect as cross-contamination. In this case, we do not need assumption $x_{\mathrm{i}}^* \approx x_{\mathrm{i}}$, since $x_1 = x_2 \equiv 1$. In addition, in the Poissonian case of the SLAMseq, the total sum of reads $X_{\mathrm{L}} + X_{\mathrm{U}}$ contains all the information about μ, and the estimate of μ is not affected by read misclassification, i.e. $\mu^* = \mu$.
For very slow genes ($\delta t \ll 1$) using the Taylor expansion for $\delta t \to 0$ it can be simplified as

$$\begin{aligned} \frac{\delta^*}{\delta} &= -\frac{1}{\delta t} \ln(1 - \delta t + o(\delta t) + \gamma(1 - 2 + 2\delta t + o(\delta t))) \\ &= -\frac{1}{\delta t} \ln(1 - \gamma + (2\gamma - 1)\delta t + o(\delta t)) \end{aligned} \tag{12}$$

For $\delta t \ll \gamma$,

$$\frac{\delta^*}{\delta} \approx -\frac{1}{\delta t} \ln(1 - \gamma) \gg 1. \tag{13}$$

In contrast, for very fast genes, such as $\delta t \gg 1$,

$$\frac{\delta^*}{\delta} \approx -\frac{\ln\gamma}{\delta t} \ll 1 \tag{14}$$

Hence, the rate estimations are biased to faster rates in the case of slow genes and toward faster values in the case of fast genes. The same result holds for the labeled fraction.

2.2 Bias due to variation in uridine content

Efficiency of biochemical separation may depend on the uridine content of the RNA species: molecules with higher uridine number are captured with higher probability. In the SLAMseq case, probability of at least one conversion event is higher for reads with high uridine content. This difference in uridine content may introduce a bias in rate estimates. To account for this bias, Eser et al. [2016] and Miller et al. [2011] introduced an additional parameter into the model.

For illustration, let us consider a labeling experiment with read numbers X_{L} (labeled fraction) available at several time points. If the probability for a molecule to be captured varies between species, we can describe it with an additional factor u:

$$\mathbb{E}X_{\mathrm{L}} = u \cdot x_2\mu(1 - e^{-\delta t}). \tag{15}$$

For RNA species, which have lower probability to be captured, than the majority of the pool (if normalization is derived from the model fitting), $u < 1$. If the normalization is done using external spike-in molecules, $u < 1$ for the species, which have lower capture probability in comparison to the labeled spike-ins. For RNA species with higher capture probability, $u > 1$.

Under the model, which does not account for the bias,

$$\mathbb{E}X_{\mathrm{L}} = x_2\mu^*(1 - e^{-\delta^* t}) = u \cdot x_2\mu(1 - e^{-\delta t}). \tag{16}$$

The distribution of the steady state levels in the labeled fraction is different than in the total fraction: the separation procedure favors species with higher uridine content:

$$\lim_{t\to\infty} \mu^*(1 - e^{-\delta t}) = u\mu \neq \mu. \tag{17}$$

Let us assume, that x_2 is derived using external spike-ins, and does not depend on the model fit. If only the labeled fraction is measured, accounting for the factor u or shifting expression level by coefficient u, such that $\mu^* = u\mu$, results in the same model fit and does not affect estimates of the degradation rate.

However, if there is data available, which allows us to better estimate μ (so $\mu^* \approx \mu$), for example, via measuring the total fraction, the bias will be introduced to the δ estimates. Indeed, for $\mu^* \approx \mu$,

$$\mathbb{E}X_{\mathrm{L}} = x_2\mu(1 - e^{-\delta^* t}) = u \cdot x_2\mu(1 - e^{-\delta t}) \tag{18}$$

$$(1 - e^{-\delta^* t}) = u(1 - e^{-\delta t}), \tag{19}$$

and $\delta^* > \delta$ if $u > 1$, and $\delta^* < \delta$ for $u < 1$.

This result is not universal, and for the case of chase experiment the relation will be the opposite: species with $u < 1$ will have lower read counts, than one would expect without accounting for the uridine bias, which shifts rate estimates towards faster degradation, i.e. for $u < 1$, $\delta^* > \delta$.

3 Fisher information matrix derivation

Let $m(\boldsymbol{\theta}, t)$ is the expected mean read number at time point t and $\boldsymbol{\theta} = (\mu, d)^T$ is a vector of model parameters.

The observed Fisher information matrix (FIM) is defined as

$$I_{\mathrm{ij}}(\boldsymbol{\theta}, X) = -\frac{\partial^2 \log \mathcal{L}(\boldsymbol{\theta}, X)}{\partial\theta_i \partial\theta_j} \tag{20}$$

In the optimal design of experiments, the expected FIM is used

$$\mathcal{I}_{\mathrm{ij}}(\boldsymbol{\theta}) = \mathbb{E} I_{\mathrm{ij}} = -\mathbb{E}\frac{\partial^2 \log \mathcal{L}(\boldsymbol{\theta}, X)}{\partial\theta_i \partial\theta_j}, \tag{21}$$

since we may not have any measurements and are interested in the performance of a given design in average. We refer everywhere only to the expected FIM and name it just FIM, omitting the "expected" term.

We will use here the fact, that the variance of the score function

$$\mathrm{var}_\theta(S(\boldsymbol{\theta})) = \mathcal{I}(\boldsymbol{\theta}), \tag{22}$$

where

$$S(\boldsymbol{\theta}) = \begin{pmatrix} \partial \log \mathcal{L}/\partial\theta_1 \\ \vdots \\ \partial \log \mathcal{L}/\partial\theta_p \end{pmatrix}. \tag{23}$$

$$S_i(\boldsymbol{\theta}) = x\frac{k}{m(m+k)}\frac{\partial m}{\partial\theta_i} + f(m,k), \tag{24}$$

where $f(m,k)$ is a term not depending on x. Using the fact that $\mathrm{var}(X) = m(m+k)/k$,

$$\mathcal{I}(\boldsymbol{\theta}) = \mathrm{var}_\theta\left(S(\boldsymbol{\theta})\right) = \frac{k}{m(m+k)}\frac{\partial m}{\partial\theta_i}\frac{\partial m}{\partial\theta_j} \tag{25}$$

In the $k \to \infty$ case, when no overdispersion is assumed and the model follows the Poisson distribution,

$$\mathcal{I}_{\mathrm{Pois}}(\boldsymbol{\theta}) = \mathrm{var}_\theta\left(S(\boldsymbol{\theta})\right) = \frac{1}{m}\frac{\partial m}{\partial\theta_i}\frac{\partial m}{\partial\theta_j} \tag{26}$$

By plugging the relations for the RNA amount $m(t) = \mu e^{-\delta t}$ (the unlabeled fraction) and $m(t) = \mu(1-e^{-\delta t})$ (the labeled fraction) in Equation 25,

$$(\mathcal{I}_{\mathrm{L}}(\boldsymbol{\theta}))_{\delta\delta} = \frac{t^2 e^{-2\delta t}\mu}{1-e^{-\delta t}}\frac{1}{1+\frac{\mu}{k}\left(1-e^{-\delta t}\right)} \tag{27}$$

$$(\mathcal{I}_{\mathrm{U}}(\boldsymbol{\theta}))_{\delta\delta} = t^2 e^{-\delta t}\mu\frac{1}{1+\frac{\mu}{k}e^{-\delta t}} \tag{28}$$

To compute and validate the expressions for the elements of the Fisher matrix, we used wxMaxima [2015] interface to the Maxima [2014] software. The supporting file is provided together with the source code of the experiment analysis: https://github.com/dieterich-lab/DesignMetabolicRNAlabeling

For the SLAMseq case,

$$\begin{aligned}\mathcal{I}_{\mathrm{slam}}(\boldsymbol{\theta}) &= \mathcal{I}_{\mathrm{L}}(\boldsymbol{\theta}) + \mathcal{I}_{\mathrm{U}}(\boldsymbol{\theta}) \\ &= \begin{pmatrix} \frac{k\mu t^2 e^{-\delta t}}{\mu e^{-\delta t}+k} + \frac{k\mu t^2 e^{-2\delta t}}{\left(\mu\left(1-e^{-\delta t}\right)+k\right)\left(1-e^{-\delta t}\right)} & \frac{kte^{-\delta t}}{\mu\left(1-e^{-\delta t}\right)+k} - \frac{kte^{-\delta t}}{\mu e^{-\delta t}+k} \\ \frac{kte^{-\delta t}}{\mu\left(1-e^{-\delta t}\right)+k} - \frac{kte^{-\delta t}}{\mu e^{-\delta t}+k} & \frac{ke^{-\delta t}}{\mu\left(\mu e^{-\delta t}+k\right)} + \frac{k\left(1-e^{-\delta t}\right)}{\mu\left(\mu\left(1-e^{-\delta t}\right)+k\right)} \end{pmatrix}\end{aligned} \tag{29}$$

The inverse matrix in the general case is

$$\mathcal{I}^{-1}_{\mathrm{slam}}(\boldsymbol{\theta}) = \begin{pmatrix} \frac{e^{\delta t}-1}{\mu t^2} + \frac{2(1-e^{-\delta t})^2}{kt^2} & \frac{e^{-2\delta t}\left(2\mu - 3\mu e^{\delta t} + \mu e^{2\delta t}\right)}{kt} \\ \frac{e^{-2\delta t}\left(2\mu - 3\mu e^{\delta t} + \mu e^{2\delta t}\right)}{kt} & \mu + \frac{\mu^2}{k}\left(e^{-2\delta t} + \left(1-e^{-\delta t}\right)^2\right) \end{pmatrix} \tag{30}$$

A model with overdispersion imposes an upper bound on the $(\mathcal{I}_{\text{slam}}^{-1}(\boldsymbol{\theta}))_{\delta\delta}$, which cannot be improved by increase of sequencing depth (i.e. μ) alone. For a fixed t,

$$\lim_{\mu\to\infty}(\mathcal{I}_{\text{slam}}^{-1}(\boldsymbol{\theta}))_{\delta\delta} = \frac{2(1-e^{-\delta t})^2}{kt^2} \tag{31}$$

Although this limit value decreases with $t \to \infty$, the depth μ must increase exponentially $e^{\delta t}/\mu(t) \to 0$ in order to satisfy

$$\lim_{t\to\infty}\frac{e^{\delta t}-1}{\mu(t)t^2} = 0. \tag{32}$$

If the total depth of n repetitions is fixed to some value such, that $n\mu_1 = \mu$, where μ_1 corresponds to the depth in a single repetition,

$$\lim_{\substack{n\to\infty \\ \mu_1 n=\mu}}\frac{1}{n}\left(\frac{e^{\delta t}-1}{\mu_1 t^2} + \frac{2(1-e^{-\delta t})^2}{kt^2}\right) = \frac{e^{\delta t}-1}{\mu t^2}, \tag{33}$$

which coincides with the FIM term for the Poissonian (non-overdispersed) model, compare to Equation 9 in the main document. Hence, it might be more beneficial to spread the sequencing capacity over several replicates.

3.1 Poissonian (no overdispersion) case

In the limit case of low overdispersion, $k \to \infty$, the FIMs for the labeled and unlabeled parts are

$$\mathcal{I}_{\text{U}}(\boldsymbol{\theta}) = \begin{pmatrix} \mu t^2 e^{-\delta t} & -te^{-\delta t} \\ -te^{-\delta t} & \dfrac{e^{-\delta t}}{\mu} \end{pmatrix} \tag{34}$$

$$\mathcal{I}_{\text{L}}(\boldsymbol{\theta}) = \begin{pmatrix} \dfrac{\mu t^2}{e^{2\delta t}-e^{\delta t}} & te^{-\delta t} \\ te^{-\delta t} & \dfrac{1-e^{-\delta t}}{\mu} \end{pmatrix} \tag{35}$$

and the total is

$$\mathcal{I}_{\text{slam}}(\boldsymbol{\theta}) = \mathcal{I}_{\text{U}}(\boldsymbol{\theta}) + \mathcal{I}_{\text{L}}(\boldsymbol{\theta}) = \begin{pmatrix} \dfrac{\mu t^2}{e^{\delta t}-1} & 0 \\ 0 & \dfrac{1}{\mu} \end{pmatrix}, \tag{36}$$

and the inverse of the FIM is simplified to

$$\mathcal{I}_{\text{slam}}^{-1}(\boldsymbol{\theta}) = \begin{pmatrix} \dfrac{e^{\delta t}-1}{\mu t^2} & 0 \\ 0 & \mu \end{pmatrix}. \tag{37}$$

3.2 Optimal time for SLAMseq

One may be interested to minimize the relative variance for the degradation rate estimator, i.e.

$$\frac{\text{var}(\hat{\delta})}{\delta^2} \approx \frac{1}{(\mathcal{I}_{\text{slam}}(\boldsymbol{\theta}))_{\delta\delta}\delta^2} \tag{38}$$

That means, that we are to maximize the denominator $(\mathcal{I}_{\text{slam}}(\boldsymbol{\theta}))_{\delta\delta}\delta^2$ term. This expression can be simplified by introducing dimensionless variable $\alpha = \delta t = t/\tau$, where τ is the characteristic time of the degradation process.

$$(\mathcal{I}_{\text{slam}}(\boldsymbol{\theta}))_{\delta\delta}\delta^2 = \frac{\mu(\delta t)^2}{e^{\delta t}-1} = \frac{\mu\alpha^2}{e^{\alpha}-1} \tag{39}$$

The optimal value of α, which maximizes the $(\mathcal{I}_{\text{slam}}(\boldsymbol{\theta}))_{\delta\delta}\delta^2$ can be derived from condition of zero derivative:

$$\begin{aligned}&\left(\frac{\mu\alpha^2}{e^\alpha - 1}\right)' = \frac{2\mu\alpha}{e^\alpha - 1} - \frac{\mu\alpha^2 e^\alpha}{(e^\alpha - 1)^2} = 0\\&(2-\alpha)e^\alpha = 2\end{aligned} \tag{40}$$

The solution can be found numerically:

$$\alpha^*_{\text{slam}} \approx 1.5936, \tag{41}$$

and for a given degradation rate δ, the optimal time in the SLAMseq setup and Poissonian assumptions is

$$t_{\text{slam}} \approx 1.59/\delta = 1.59\tau. \tag{42}$$

3.3 Partial conversion case and pulse-chase SLAMseq

If only some fraction of the nascent RNA is classified as labeled, for example, the $T \to C$ conversion is not 100% efficient, the saturation level of the labeled molecules will be less than the mean expression level μ:

$$\text{pulse}\begin{cases} m_{\text{U}}(t) = \mu^*_1 + \mu^*_2 e^{-\delta t} \\ m_{\text{L}}(t) = \mu^*_2(1 - e^{-\delta t}). \end{cases} \tag{43}$$

In this model, the unlabeled fraction never extincts. In the steady-state, after long enough labeling, the unlabeled fraction approaches its minimal level μ^*_1, and the labeled one reaches its maximum μ^*_2, where $\mu = \mu^*_1 + \mu^*_2$.

In the case of the pulse-chase design, which we analyze in this work, the labeled fraction degrades starting from some initial level $\mu_2 \leqslant \mu^*_2$, and if the system is close to the steady state after the pulse-phase, $\mu_2 \approx \mu^*_2$ and $\mu_1 \approx \mu^*_1$:

$$\text{chase}\begin{cases} m_{\text{U}}(t) = \mu_1 + \mu_2(1 - e^{-\delta t}) \\ m_{\text{L}}(t) = \mu_2 e^{-\delta t}, \end{cases} \tag{44}$$

with $\mu = \mu_1 + \mu_2$.

If there is only one time point measured, it is not possible to identify uniquely all three parameter (δ, μ_1 and μ_2) of the algebraic system. For example, additional measurement at the beginning of the chase phase at $t = 0$ would provide direct information on μ_1 and μ_2, since $m_{\text{U}}(0) = \mu_1$ and $m_{\text{L}}(0) = \mu_2$.

Presence of the background level in the unlabeled fraction μ_1 negatively affects our estimations. In the limiting case, when the background level μ_1 is very large $\mu_1 \gg \mu_2$, the unlabeled fraction hardly contributes any information on our parameter of the interest δ. More formally, considering the pulse-chase design (eq. 44) under the Poissonian assumptions (eq. 26), the diagonal element

$$(\mathcal{I}_U(\boldsymbol{\theta}))_{\delta\delta} = \frac{1}{m}\left(\frac{\partial m}{\partial \delta}\right)^2 = \frac{\mu_2 e^{-2\delta t} t^2}{\mu_1/\mu_2 + (1 - e^{-dt})} \tag{45}$$

$$(\mathcal{I}_L(\boldsymbol{\theta}))_{\delta\delta} = \mu_2 t^2 e^{-\delta t}, \tag{46}$$

and if the fraction of the molecules with conversion is very small in comparison to the background level μ_1,

$$\lim_{\mu_1/\mu_2 \to \infty} (\mathcal{I}_U(\boldsymbol{\theta}))_{\delta\delta} = 0. \tag{47}$$

The term for the labeled fraction $(\mathcal{I}_L(\boldsymbol{\theta}))_{\delta\delta}$ does not depend on the level of background counts μ_1. Hence, in the limiting case of high background level $\mu_1/\mu_2 \to \infty$, the main contribution to the $(\mathcal{I}_{\text{slam}}(\boldsymbol{\theta}))_{\delta\delta} = (\mathcal{I}_U(\boldsymbol{\theta}))_{\delta\delta} + (\mathcal{I}_L(\boldsymbol{\theta}))_{\delta\delta}$ comes from $(\mathcal{I}_L(\boldsymbol{\theta}))_{\delta\delta}$. Since $(\mathcal{I}_L(\boldsymbol{\theta}))_{\delta\delta} = \mu_2 t^2 e^{-\delta t}$ has its maximum at $t = 2/\delta = 2\tau$, in the limiting case of $\mu_1/\mu_2 \to \infty$, the maximum of the $(\mathcal{I}_{\text{slam}}(\boldsymbol{\theta}))_{\delta\delta}$ is shifted towards $t = 2\tau$. It is to be compared to the pulse-only experiment or pulse-chase started from a steady state SLAMseq, in which case optimal time is $t \approx 1.59\tau$ ($\mu_1 = 0$).

In the result section on the SLAMseq experiment analysis, we illustrate the dependency of the $(\mathcal{I}_{\text{slam}}(\boldsymbol{\theta}))_{\delta\delta}$ on the labeling time t without Poissonian assumptions. For completeness, we provide here the expression for this case as well:

$$(\mathcal{I}_{\text{slam}}(\boldsymbol{\theta}))_{\delta\delta} = \frac{k\mu_2 t^2 e^{-\delta t}}{\mu_2 e^{-\delta t} + k} + \frac{k{\mu_2}^2 t^2 e^{-2\delta t}}{(\mu_2(1 - e^{-\delta t}) + \mu_1)(\mu_2(1 - e^{-\delta t}) + \mu_1 + k)}. \tag{48}$$

3.4 On the alternative parametrization

If one considers the logarithm of the degradation rate as a parameter for the statistical model, i.e. $\eta = \ln(\delta)$ and $\delta(\eta) = e^{\eta}$, then the Fisher information matrix term

$$\mathcal{I}_{\eta\eta}(\boldsymbol{\theta}) = \mathcal{I}_{\delta\delta}(\boldsymbol{\theta}) \left| \frac{\mathrm{d}\delta(\eta)}{\mathrm{d}\eta} \right|^2 = \mathcal{I}_{\delta\delta}(\boldsymbol{\theta}) e^{2\eta} = \mathcal{I}_{\delta\delta}(\boldsymbol{\theta})\delta^2, \tag{49}$$

which coincides with the modified term $\mathcal{I}_{\delta\delta}(\boldsymbol{\theta})\delta^2$, which we used to optimize the relative variance $\mathrm{var}(\hat{\delta})/\delta^2$ in the main text. Although $\mathcal{I}_{\eta\eta}(\boldsymbol{\theta})$ alone may look less cumbersome, we avoided introducing additional parameters and stick to the usage of the initial parameter δ only.

3.5 Asymptotics at extreme labeling times

Here we investigate the behaviour of the relative variance at the times, which are much shorter ($\alpha \to 0$) and much longer ($\alpha \to \infty$), than the characteristic time τ for a given gene. Since $\mathcal{I}_{\mathrm{slam}}(\boldsymbol{\theta}) = \mathcal{I}_{\mathrm{U}}(\boldsymbol{\theta}) + \mathcal{I}_{\mathrm{L}}(\boldsymbol{\theta})$,

$$\begin{aligned}
(\mathcal{I}_{\mathrm{U}}(\boldsymbol{\theta}))_{\delta\delta}\delta^2 &= \mu(\delta t)^2 e^{-\delta t} = \mu\alpha^2 e^{-\alpha} \\
(\mathcal{I}_{\mathrm{L}}(\boldsymbol{\theta}))_{\delta\delta}\delta^2 &= \frac{\mu(\delta t)^2}{e^{2\delta t} - e^{\delta t}} = \frac{\mu\alpha^2}{e^{2\alpha} - e^{\alpha}} \\
(\mathcal{I}_{\mathrm{slam}}(\boldsymbol{\theta}))_{\delta\delta}\delta^2 &= \frac{\mu\alpha^2}{e^{\alpha} - 1}
\end{aligned} \tag{50}$$

- $\alpha \to 0$
  Using the Taylor expansion, $e^{\alpha} = 1 + \alpha + o(\alpha)$, where $\lim_{\alpha \to 0} o(\alpha)/\alpha = 0$,

$$\begin{aligned}
(\mathcal{I}_{\mathrm{U}}(\boldsymbol{\theta}))_{\delta\delta}\delta^2 &= \mu\alpha^2(1 - \alpha + o(\alpha)) \sim \alpha^2 \\
(\mathcal{I}_{\mathrm{L}}(\boldsymbol{\theta}))_{\delta\delta}\delta^2 &= \frac{\mu\alpha^2}{1 + 2\alpha - 1 - \alpha + o(\alpha)} \sim \alpha \\
(\mathcal{I}_{\mathrm{slam}}(\boldsymbol{\theta}))_{\delta\delta}\delta^2 &= ((\mathcal{I}_{\mathrm{U}}(\boldsymbol{\theta}))_{\delta\delta} + (\mathcal{I}_{\mathrm{L}}(\boldsymbol{\theta}))_{\delta\delta})\delta^2 \sim \alpha
\end{aligned} \tag{51}$$

- $\alpha \to \infty$
  Since $\lim_{x \to \infty} \log(x)/x = 0$,

$$\begin{aligned}
(\mathcal{I}_{\mathrm{U}}(\boldsymbol{\theta}))_{\delta\delta}\delta^2 &= \mu e^{-\alpha(1 + 2\log(\alpha)/\alpha)} \sim e^{-\alpha} \\
(\mathcal{I}_{\mathrm{L}}(\boldsymbol{\theta}))_{\delta\delta}\delta^2 &= \mu e^{-2\alpha + 2\log(\alpha) - \log(1 - e^{-\alpha})} \sim e^{-2\alpha} \\
(\mathcal{I}_{\mathrm{slam}}(\boldsymbol{\theta}))_{\delta\delta}\delta^2 &= (\mathcal{I}_{\mathrm{U}}(\boldsymbol{\theta}))_{\delta\delta}\delta^2 + (\mathcal{I}_{\mathrm{L}}(\boldsymbol{\theta}))_{\delta\delta}\delta^2 \sim e^{-\alpha}
\end{aligned} \tag{52}$$

  This result shows, that relative variance of the $\hat{\delta}$ estimator increases exponentially at very long labeling times ($t \gg \tau$), whereas at short times ($t \ll \tau$) it behaves as a power function.

4 Normalization in conventional experiments

The situation is different for the case of biochemical purification, when the labeled, unlabeled and total fractions are sequenced to the approximately same depth. For the next derivation, we assume that samples were normalized externally, e.g. by synthetic spike-ins or exogenous RNA. In addition, we do not consider uncertainty coming from fraction normalization.

The sequencing depth for the total sample is $\sum_i \mu_i$. After labeling for t hr, the concentrations of labeled and unlabeled molecules changes according to Equation 9, and in the case of same depth, the normalization coefficients are

$$x_{\mathrm{L}} \approx \frac{\sum_i \mu_i}{\sum_i \mu\,(1 - e^{-\delta_i t})} \tag{53}$$

$$x_{\mathrm{U}} \approx \frac{\sum_i \mu_i}{\sum_i \mu_i e^{-\delta_i t}}, \tag{54}$$

where we use the total sample as a reference, i.e. with the normalization coefficient 1. At long times, when majority of genes in the labeled fraction achieve saturation, $x_{\mathrm{L}} \approx 1$. Similarly, at short times, $x_{\mathrm{U}} \approx 1$, degradation has a minor effect on the unlabeled fraction.

In contrast, at short times,

$$x_{\mathrm{L}} \approx \frac{\sum_i \mu_i}{\sum_i \mu \delta_i t} = \frac{1}{\langle \delta \rangle t}, \tag{55}$$

where $\langle \delta \rangle = \sum_i \mu_i \delta_i / \sum_i \mu_i$ is average degradation rate weighted by the steady-state expression level. If there is a small cluster of fast genes $i \in \mathcal{F}$, which dominate the pool of labeled molecules at such times, that $(1 - e^{-\delta_i t}) \ll 1$ for other genes ($i \notin \mathcal{F}$), and $(1 - e^{-\delta_i t}) \approx 1$ for $i \in \mathcal{F}$, then

$$x_{\mathrm{L}} \approx \frac{\sum_i \mu_i}{\sum_{i \in \mathcal{F}} \mu_i}. \tag{56}$$

Similar result holds true for the cluster of slow genes $\mathcal{S}$, such that $e^{-\delta_i t} \approx 1$ for $i \in \mathcal{S}$ and $e^{-\delta_i t} \ll 1$ for $i \notin \mathcal{S}$, and

$$x_{\mathrm{U}} \approx \frac{\sum_i \mu_i}{\sum_{i \in \mathcal{S}} \mu_i}. \tag{57}$$

Such "zooming" effect of normalization can drastically improve inference about fast genes in comparison to the SLAMseq design, since x_{L} and x_{U} can be interpreted as a corresponding increase in depth in SLAMseq experiment.

Modifying the mean read count in Equations 27 and 28 by the factors x_{L} and x_{U} results in

$$(\mathcal{I}_{\mathrm{L}}(\boldsymbol{\theta}))_{\delta\delta} = \frac{t^2 e^{-2\delta t}}{(1 - e^{-\delta t})} \frac{1}{\frac{1}{x_{\mathrm{L}} \mu} + \frac{1}{k}(1 - e^{-\delta t})} \tag{58}$$

$$(\mathcal{I}_{\mathrm{U}}(\boldsymbol{\theta}))_{\delta\delta} = t^2 e^{-\delta t} \frac{1}{\frac{1}{x_{\mathrm{U}} \mu} + \frac{1}{k} e^{-\delta t}} \tag{59}$$

As in the SLAMseq case, the overdispersion imposes limits on the terms of the FIM:

$$\lim_{\mu \to \infty} (\mathcal{I}_{\mathrm{L}}(\boldsymbol{\theta}))_{\delta\delta} = \frac{t^2 e^{-2\delta t} k}{(1 - e^{-\delta t})^2} \leqslant \frac{k}{\delta^2} \tag{60}$$

$$\lim_{\mu \to \infty} (\mathcal{I}_{\mathrm{U}}(\boldsymbol{\theta}))_{\delta\delta} = t^2 k. \tag{61}$$

It is interesting, that in the case of the unlabeled fraction, this upper bound can be improved by using longer labeling times, which is not true for the labeled one.

References

Simon Anders and Wolfgang Huber. Differential expression of RNA-Seq data at the gene level–the deseq package. *Heidelberg, Germany: European Molecular Biology Laboratory (EMBL)*, 2012.

Philipp Eser, Leonhard Wachutka, Kerstin C Maier, Carina Demel, Mariana Boroni, Srignanakshi Iyer, Patrick Cramer, and Julien Gagneur. Determinants of RNA metabolism in the schizosaccharomyces pombe genome. *Molecular systems biology*, 12(2):857, 2016.

Maxima. Maxima, a computer algebra system. version 5.34.1, 2014. URL http://maxima.sourceforge.net/.

Christian Miller, Björn Schwalb, Kerstin Maier, Daniel Schulz, Sebastian Dümcke, Benedikt Zacher, Andreas Mayer, Jasmin Sydow, Lisa Marcinowski, Lars Dölken, et al. Dynamic transcriptome analysis measures rates of mRNA synthesis and decay in yeast. *Molecular systems biology*, 7(1):458, 2011.

wxMaxima. wxMaxima, a computer algebra system. version 15.08.2, 2015. URL http://andrejv.github.io/wxmaxima/.
