# Supplementary figures and images for: On the optimal design of metabolic RNA labeling experiments
Source: PLoS Comput Biol. 2019 Aug 7;15(8):e1007252. doi: 10.1371/journal.pcbi.1007252 (PMC6699717; doi:10.1371/journal.pcbi.1007252)

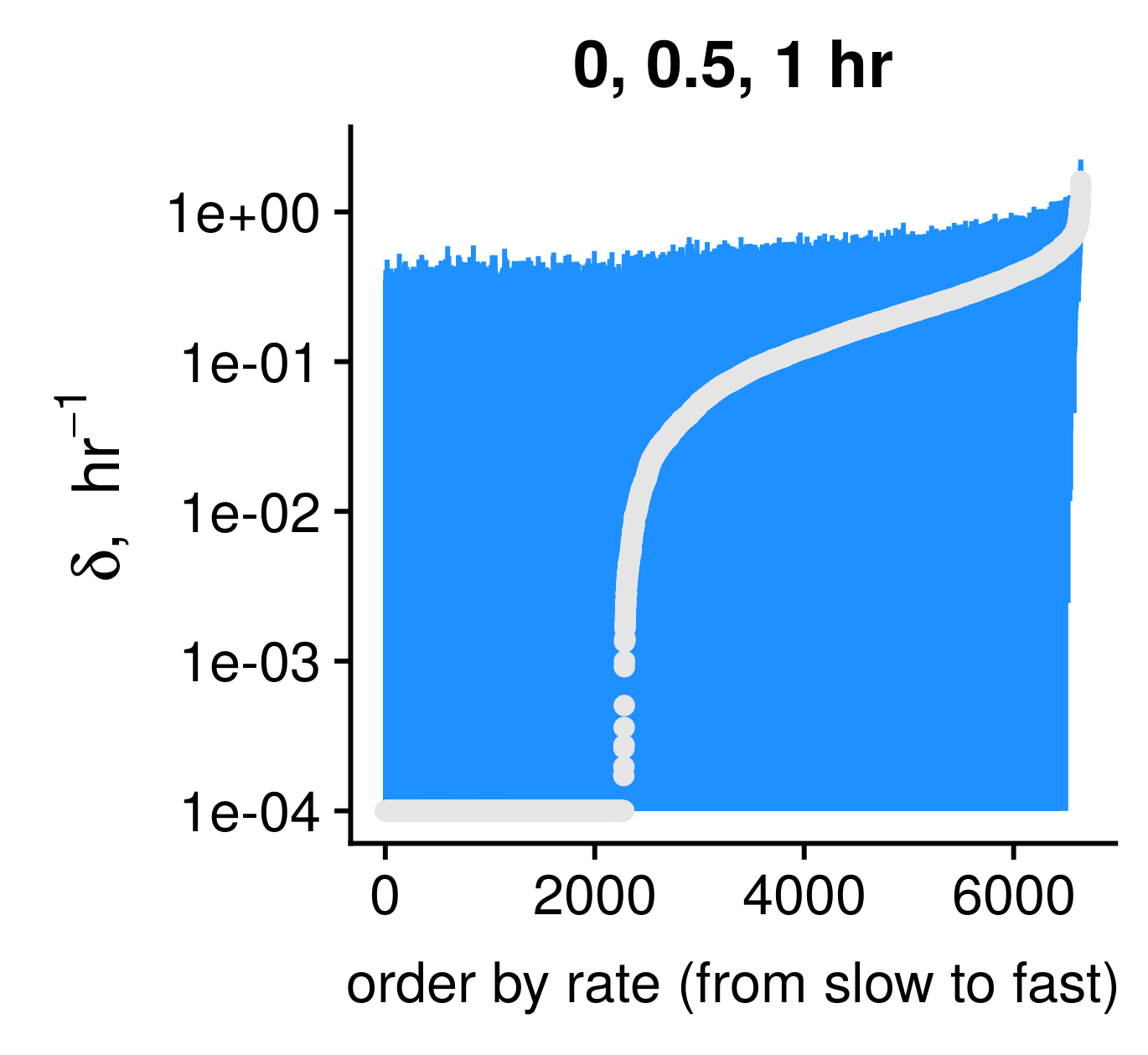

Supplement: S1 Fig — 95% profile likelihood confidence intervals for the estimates of the degradation rate δ^, derived from the time points at 0, 0.5 and 1 hr of the chase phase. (TIFF) [file pcbi.1007252.s002.tiff]

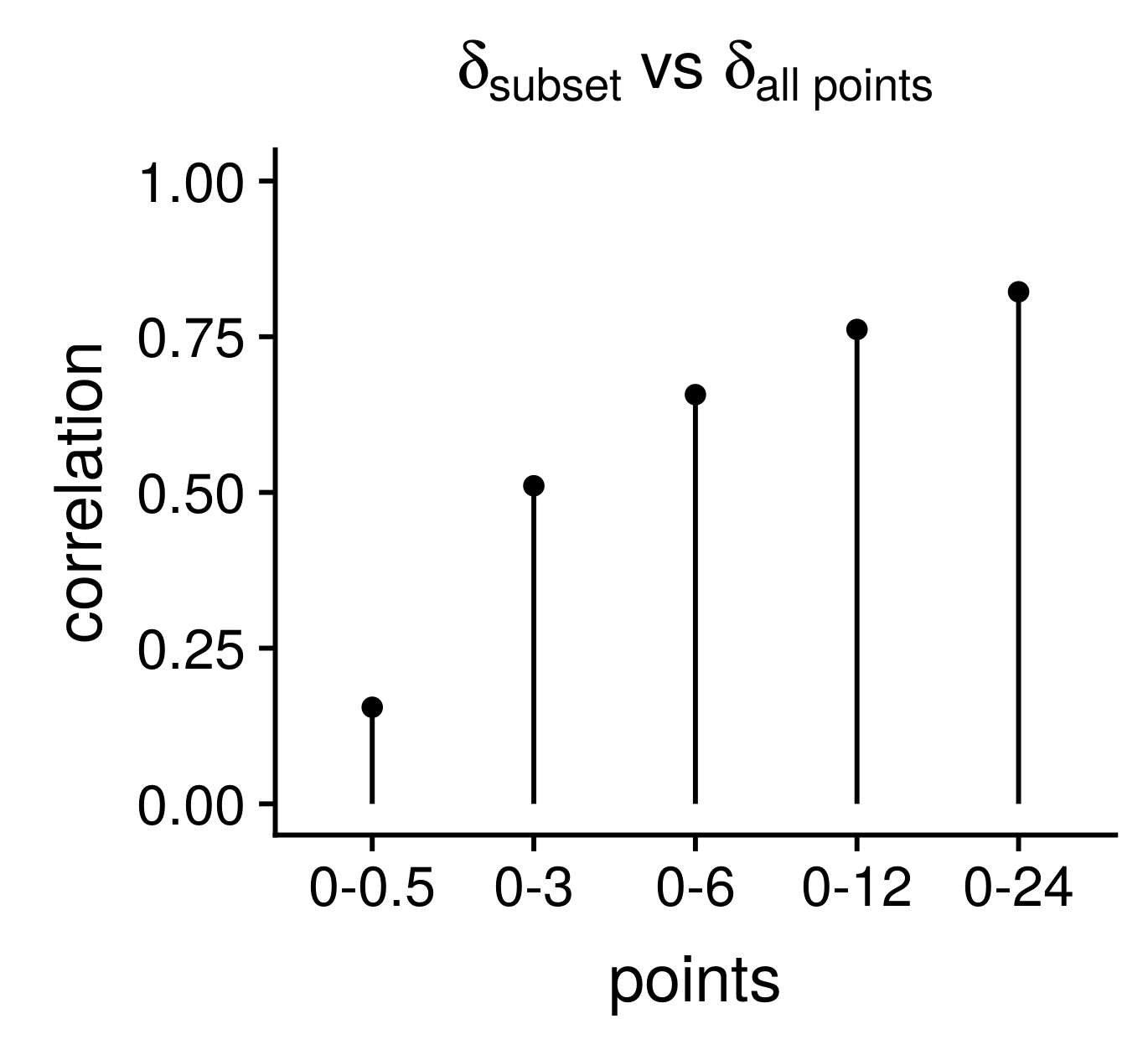

Supplement: S2 Fig — Spearman correlation between estimates of degradation rates, computed for different subsets of time points, and the rates derived from the whole data set. (TIFF) [file pcbi.1007252.s003.tiff]

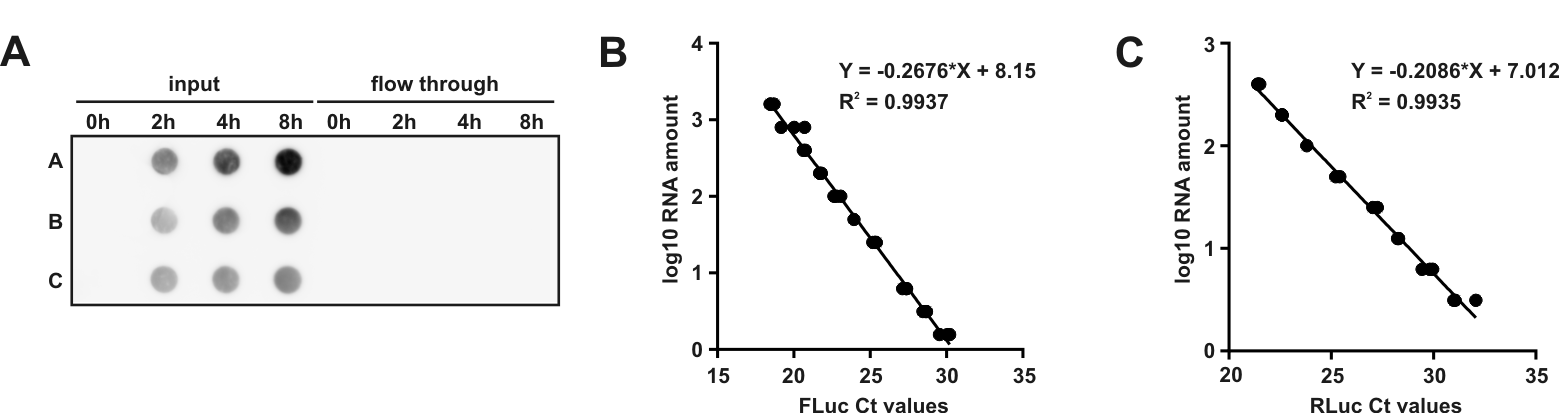

Supplement: S3 Fig — A: Dot blot-based detection of biotinylation with streptavidin-HRP in input and flow through of streptavidin purification from three replicate experiments A-C. The quantification of the captured image is shown in Fig 3A. B: Standard curve for the absolute quantification of 4sU-labeled FLuc RNA. 1600 to 1.56% of the input used for streptavidin purification was measured by RT-qPCR analysis in 1:2 dilutions. The log10 amount of RNA was plotted against the obtained Ct value and used for linear regression. C: Standard curve for the absolute quantification of unlabeled RLuc RNA. 400 to 3.13% of the input used for streptavidin purification was measured by RT-qPCR analysis in 1:2 dilutions. The log10 amount of RNA was plotted against the obtained Ct value and used for linear regression. (TIFF) [file pcbi.1007252.s004.tiff]
